# Smart patterned surfaces with programmable thermal emissivity and their design through combinatorial strategies

**DOI:** 10.1038/s41598-017-13132-6

**Published:** 2017-10-10

**Authors:** N. Athanasopoulos, N. J. Siakavellas

**Affiliations:** Department of Mechanical Engineering & Aeronautics, University of Patras, 26500 Patras, Greece

## Abstract

The emissivity of common materials remains constant with temperature variations, and cannot drastically change. However, it is possible to design its entire behaviour as a function of temperature, and to significantly alter the thermal emissivity of a surface through the combination of different patterns and materials. We show that smart patterned surfaces consisting of smaller structures (motifs) may be designed to respond uniquely through combinatorial strategies by transforming themselves. The smart surfaces can passively manipulate thermal radiation—without the use of electronics—because their *modus operandi* has already been programmed into their intrinsic characteristics; the environment provides the energy required for their activation. Each motif emits thermal radiation in a certain manner, as it changes its geometry; however, the spatial distribution of these motifs causes them to interact with each other. Therefore, their combination and interaction determine the global behaviour of the surfaces, thus enabling their *a priori* design. The emissivity behaviour is not random; it is determined by two fundamental parameters, namely the combination of orientations in which the motifs open (n-fold rotational symmetry) and the combination of materials (colours) on the motifs; these generate functions which fully determine the dependency of the emissivity on the temperature.

## Introduction

Temperature control is one of the most common processes in Nature and man-made systems. By observing the manner in which plants and animals control their temperature^1–5^ and their geometrical characteristics^6–8^, we may deduce that Nature is a specialist in purely mechanistic thermal management strategies. Nature addresses thermal management issues—prevention of overheating and damages—through an efficient holistic and mechanistic design approach. Man-made and natural strategies combine the geometrical characteristics, the materials, and the patterns of the outer surface of a body to handle the thermal energy exchange in the nano-, micro- or macro-scale.

By utilising common paints^9^ and by forming arrays in micro or in macroscale^10–14^, certain emissivity values can be achieved that would allow the temperature regulation of a body and emittance direction. On the other hand, to achieve variable thermal emissivity properties, different approaches have been adopted according to the target application, namely the development of: i) advanced materials^15–17^, ii) active metamaterials^18–20^ through the formation of patterns in microscale, iii) active micro-electromechanical systems which incorporate materials of different thermo-optical properties^21,22^, and iv) the development of systems, such as mechanical louvers^23^ and morphing radiators, which conceal/reveal materials of different emissivity values using shape memory alloys^24,25^. Furthermore, in architecture, large morph-able facades have been proposed for the shading of buildings^26–29^. The present authors have preliminary studied the feasibility to achieve variable thermal emissivity behaviour of a single self-shape structure^30^. The self-shape structures interact with their environment passively, as opposed to MEMs, which use high-voltage power supplies in order to change the effective thermal emissivity.

While the extremely heavy mechanical systems and complex MEMs use hinges, actuators in order to develop simple movements, in Nature extremely complex movements can be realized through the materials’ self-shaping & self-folding capabilities in response to a stimulus^30^. Thermonastic movements, meaning ‘folding caused by a temperature stimulus’, are realized in order to transform the shape of leaves/petals under a temperature stimulus. The view factor (geometrical characteristics of the radiative object), and the material that is exposed to the environment are regulated in Nature for prevention from overheating and damages^3,4^. More specifically, the drooping of rhododendron leaves protects them from damage due to high irradiance and cold temperatures^3^, while poplar’s leaves present dual thermal reflectivity values on both of their leaves’ surfaces for damage prevention^4^.

Based on the aforementioned mechanistic strategies, and taking them one step further, we theoretically and experimentally studied smart patterned surfaces *(in a broader context “patterned surfaces” may be referred to as “metasurfaces”)* and identified their fundamental properties, which govern the global behaviour of the overall thermal energy emission. We prove that smart patterned surfaces consisting of smaller structures (motifs) may be designed to respond uniquely through combinatorial design strategies. The combination and interaction of the motifs determine the global behaviour of the surfaces, thus enabling their a priori design of the effective thermo-optical properties as a function of temperature.

Currently, there are no studies in radiative heat transfer in which a property (i.e. emissivity) can be fully designed as a function of another parameter (temperature). In the present research work, we studied theoretically and we proved experimentally that we can design the entire emissivity function of a patterned surface through the combination of the orientation and the colour sequences of its motifs. Through this mechanistic approach and the combination of at least two different materials with different thermo-optical properties (colours) and motifs with two different orientations, entire families of different emissivity curves can be generated. The emissivity value could be significantly altered (ε_max_ ≥ 20ε_min_) passively within a small temperature change (ΔΤ ≈ 20^ο^C), and the generated functions can be classified according to their behaviour. In contrast, MEMs can be change their emissivity dynamically (ε_max_ ≥ 5ε_min_)^21,22^.

The change may either be positive or negative (Δε < 0 or Δε > 0). Moreover, these surfaces are low-weight and they interact passively with their environment. Despite the fact that this study is not directly correlated with other types of metamaterials (mechanical metamaterials), it is important to be mentioned that in the field of mechanical metamaterials^31–33^ and thermal metamaterials^34^, interesting studies have revealed the importance of the manner in which a value of a property can be designed using combinatorial strategies^32^. Furthermore, through the combination and the interaction of oriented unit cells^31^ of a mechanical metamaterial, it is possible to design the local or global mechanical response.

In our study, the entire function of the effective emissivity of a surface ε_eff_(T) can be designed by controlling its global maximum or minimum, its linearity, convexity, inflection point, and other characteristics. Ultimately, the handling of the effective thermo-optical properties of a surface through a material that interacts with light and temperature will lead to the development of advanced materials and structures for optimized thermal applications in satellites and other energy systems.

## Generalised Approach on a Ditranslational Lattice

**Figure 1:**
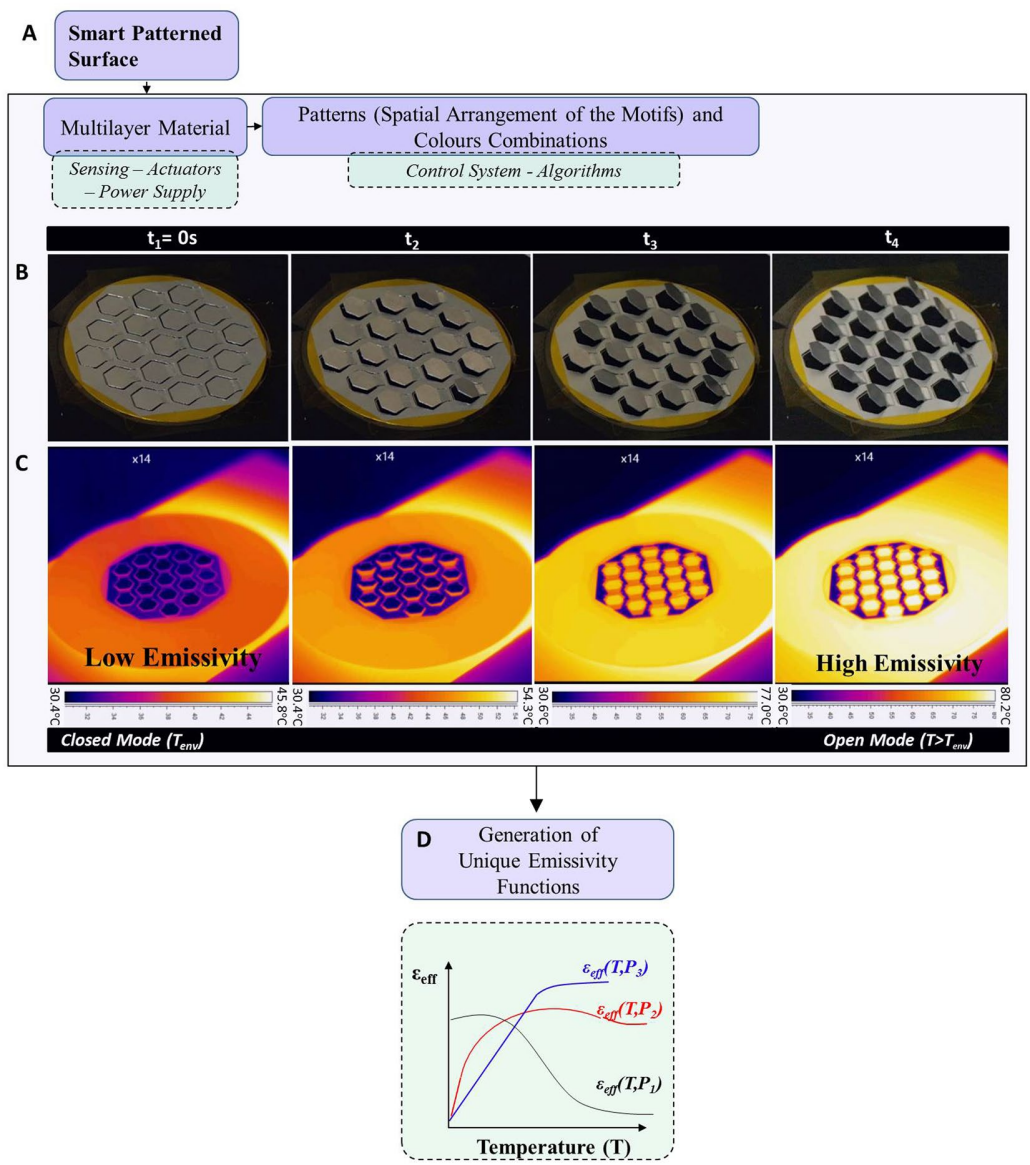
Variable and programmable emissivity through smart patterned surfaces. (**A**) Flow diagram of the *modus operandi* of the materials. (**B**) Developed patterned surface on hexagonal ditranslational lattice with variable emissivity as a function of temperature (T_env_ ≤ T ≤ 80.2 °C, Tmax ≈ 100 °C). The patterned surfaces can assume states from closed to open (Video S1) or from open to closed (Video S2). (**C**) Thermography during the heating stage. (**D**) Possible generated emissivity curves using different colour and orientation combinations.

The principle of the variable and programmable emissivity through smart patterned surfaces is illustrated in Fig. 1. The transformation of the motifs conceals (closed geometry) or reveals (open geometry) one of the materials (Fig. 1a–c), hereafter referred to as ‘colours’, and regulates the view factor of the patterned surfaces, thus enabling the realisation of a variable and programmable effective thermal emissivity (ε_eff_) (Videos S1 and S2). Our scope is to investigate how the interactions between the motifs manipulate the total thermal energy which is emitted from the patterned surfaces, and to demonstrate that it is possible to generate unique emissivity functions such as the ones illustrated in Fig. 1d. The effective emissivity of a patterned surface may increase or decrease with temperature. In Fig. 1c, using a thermal camera we may clearly observe the increase in the emissivity of the internal regions of the smart surface as the temperature increases.

### Geometrical Transformation of the Motifs

The material can be developed to passively react over a very broad range of thermal requirements (i.e. −270 °C/+350 °C), something which cannot be achieved by shape memory polymers, pre-strained polymeric sheets^35^ or shape memory alloys^36^ whose transition temperature is fixed to a limited range of phase transition temperatures. In this study, we developed anisotropic multilayer materials for which a temperature change generates internal stresses that cause the transformation of the motifs (Methods; Fig. 1, Video S4), at any temperature level. These materials behave in a similar manner to the ‘4D-biomimetic materials^37–41^. Owing to the large displacements and rotations of the multilayer material, non-linear phenomena appear^42^ (Methods, Video S4).

The transformation of the motifs is the ‘driving force’ in accomplishing our purpose; however, our scope is to investigate the impact of the interactions between the motifs on the total thermal energy which is emitted from the patterned surfaces, for all directions and wavelengths (effective total hemispherical emissivity, ε_eff_(T)).

### Fundamental parameters

**Figure 2:**
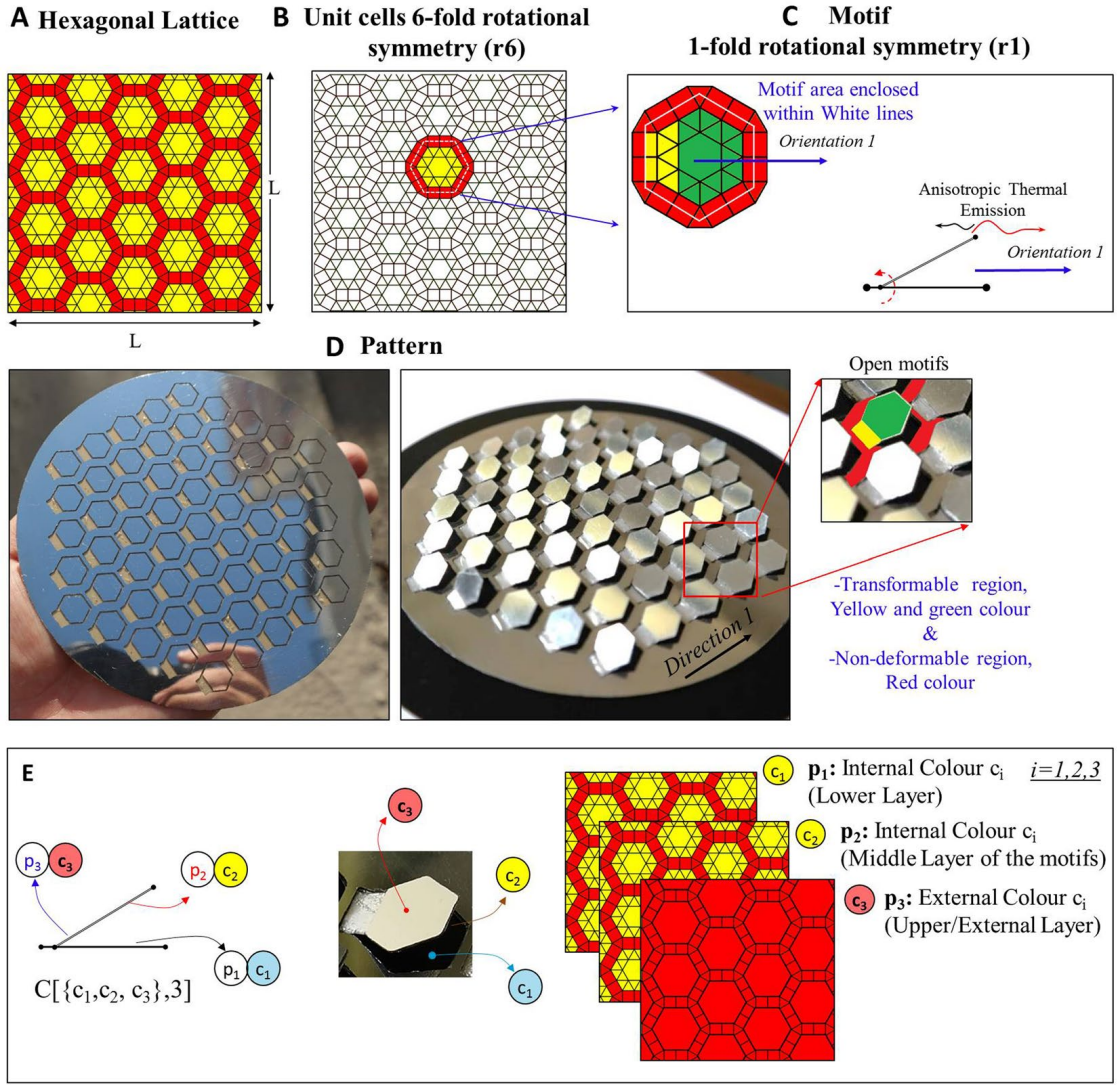
Patterned surface incorporating motifs with 1-fold rotational symmetry (r1). (**A**) Ditranslational hexagonal lattice. (**B**) 6-fold rotational symmetry unit cells. (**C**) Motif with 1-fold rotational symmetry. The possible orientations of the motifs on the ditranslational lattice are related to the rotational symmetry of the motif and of the unit cells. (**D**) Developed patterned surface (Video S3) with low emissivity on the external surface and high emissivity on the internal surfaces of the motifs and depiction of the transformable and non-deformable regions of the motifs. (**E**) Transformable region of the motif and different materials (colours, c_i_ = 1,2,3) with different emissivity values that can be applied on three different layers (possible positions). Each motif could open facing a particular direction(s), concealing/revealing the internal layers to the environment.

The effective emissivity behaviour of the patterned surface is determined by two fundamental parameters, namely a) the combination of orientations in which the motifs open and b) the combination of materials (colours) on the motifs; these generate functions which fully determine the dependency of the emissivity on the temperature. i.e. all surfaces can be represented as a ditranslational lattice, which is composed of unit cells (Fig. 2a,b). The lattice can be entirely tiled by motifs; each motif may have a different orientation on the lattice (Fig. 2c). The combination of motifs of different orientations constitutes a pattern (Fig. 2d). The behaviour of the generated emissivity curves is attributed mainly to: i. the combination (permutations) of orientations in which the motifs open or close (n-fold rotational symmetry (rn)), (Fig. 2c,d). and the ii. combination (permutations) of colours (Fig. 2e) which have been applied onto the internal and external areas (positions/layers) of the motifs. Therefore, we studied the interaction of two different ordered lists of elements with repetitions. Essentially, we focused on the order/sequence of the orientations of the motifs, the sequence of the applied colours, and their impact on the total hemispherical emissivity as a function of temperature (ε_eff_(T)), of the patterned surface (global response).

The change in the emissivity is mainly due to the coexistence of different colours which have been placed on the internal and external surfaces of the motifs (Fig. 2e), and to the interaction thereof (successive reflections and absorptions). These materials have certain emissivity and absorptivity values, and we assumed that they are grey (ε_1_ ≠ ε_2_, α_1_ ≠ α_2_, *a*_1_/*ε*_1_ = *a*_2_/*ε*_2_ = 1). Three different colours (*c*_*i*_, where i = 1, 2, 3) may be combined and placed on three different motif layers/positions (*p*), *C*[{*c*_1_,*c*_2_,*c*_3_},*p*], (Fig. 2e). The simplest case is to select two different colours, and to set them to three different layers/positions *C*[{*c*_1_,*c*_2_},3], Fig. 2d.

In addition, each motif may have a certain orientation in which it opens or closes; consequently, the motifs can anisotropically emit thermal radiation according to their rotational symmetry (1, 2, or n-fold rotational symmetry (rn)). A ditranslational hexagonal lattice^43^ may consist of unit cells of six possible different directions (Fig. 2b). In this case, 1-fold rotational symmetry (r1′) motifs (Fig. 2c) could open on a 6-fold rotational symmetry (r6) unit-cell, *P*[{1, 2, 3, ..., 6},*N*] = ((*r6*)/(*r1'*))^*N*^, where (P) is the number of possible permutations with repetitions (sequences), (N) is the number of motifs on the patterned surface, (rn) is the n-fold rotational symmetry of the unit cell, and (rn′) is the n′-fold rotational symmetry of the motif.

The major characteristics of the emissivity functions ε_eff_(T) can be controlled through the colour sequences (C) and the sequences of motifs (P) on a lattice (pattern). The sequence of the orientations of the motifs and the colours determines the maximum absolute change in emissivity (Δε_max_) for a specific temperature change (ΔΤ), as well as the ‘path’ which the emissivity follows as the temperature of the surface changes. Consequently, each sequence generates a function of ε_eff_(${{\rm{G}}}_{C}^{P}$,T) of certain characteristics, which can be manipulated through the orientation–colour coupling of these sequences.

## Tiling a Strip (Monotranslational Lattice) - Combinatorial Design

### Orientation Sequences - 1^st^ List of Elements

**Figure 3:**
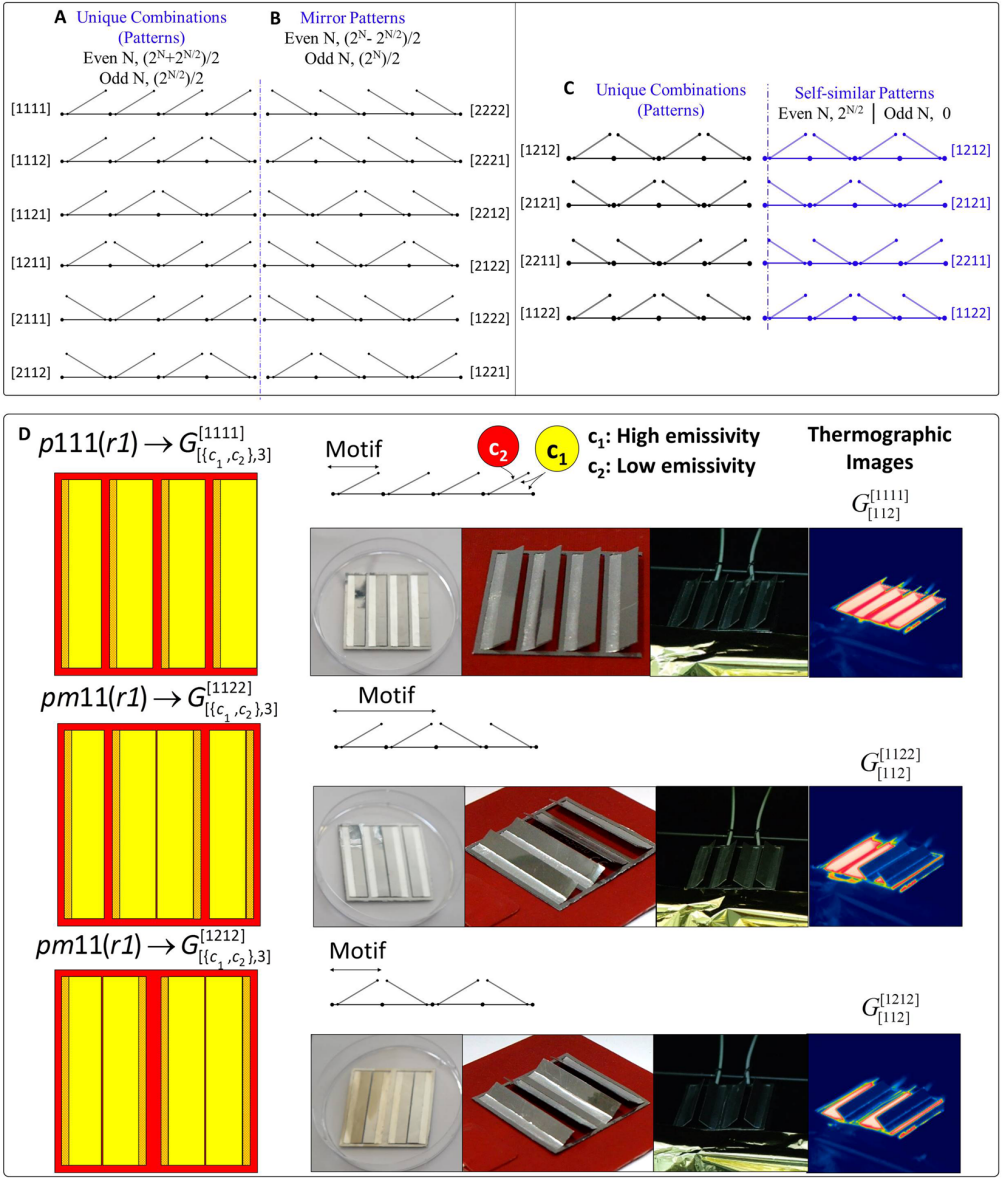
Motifs on a strip. (**A**) Unique number of permutations of a pattern with N = 4. (**B**) Generated mirror patterns. (**C**) Generated self-similar patterns. (**D**) Experimentally studied patterns (${{\rm{G}}}_{[112]}^{[1111]},{{\rm{G}}}_{[112]}^{[1122]},{{\rm{G}}}_{[112]}^{[1212]}$) during different preliminary tests (Fig. S2, Video S5).

By tiling a finite strip (mono-translational lattice) through the repetition of a motif along this strip, a variety of patterns are generated (Fig. 3a–c). The unit cell of the mono-translational lattice has a 2-fold rotational symmetry (r2). In this case, each motif with a 1-fold rotational symmetry (r1) may be oriented to face two different directions (Right and Left), such that the motif facing one direction is the mirror of the motif which faces the other direction (Fig. 3a,b).

Therefore, in the strip problem, the motifs may receive only two discrete values, namely the right (R ≡ 1) and the left (L ≡ 2). For a combination of (N) motifs with 1-fold rotational symmetry, it becomes possible to generate P[{R,L},N] ≡ P [{1,2},N] = 2^N^ sequences. If (N) is an odd number, 2 ^N^ patterns can be generated; therefore, 2^N^/2 are the mirror patterns and 2^N^/2 are the unique generated patterns. Owing to the fact that the effective properties constitute a global property which is not related to the direction of the overall pattern—however, it is related to the orientation of the motifs—all mirror symmetries of the generated patterns are equivalent to each other for any colour sequence (C). If (N) is an even number, then (2^N^ − 2^N/2^)/2 mirror patterns and 2 ^N/2^ self-similar (self-dual) mirror patterns can be generated. The self-similar patterns are the unique sequence for which the mirror of a pattern returns the exact same pattern with the exact same overall radiative behaviour (Fig. 3c). The unique generated patterns are equal to the total generated patterns minus the unique mirror patterns, P = (2^N^ + 2^N/2^)/2 (Methods).

### Colour Sequences - 2^nd^ List of Elements

Moreover, each motif may comprise a combination of colours. By applying two different colours (Fig. 3d), i.e. black (high emissivity = ε_1_ = c_1_) and silver (low emissivity = ε_2_ = c_2_), in three different available positions (p) on the layers of the motifs, the number of possible colour sequences is $C[\{{c}_{1},{c}_{2},\mathrm{..},{c}_{i}\},p]={{\rm{c}}}^{{\rm{p}}}\underset{i=2}{\overset{p=3}{\longrightarrow }}C[\{{c}_{1},{c}_{2}\},3]={2}^{3}$. The number of unique orientation and colour sequences and, consequently, the number of generated functions ε_eff_ (${{\rm{G}}}_{C}^{P}$,T) of a finite-length strip can be expressed via the following equations.

1 $$\begin{array}{l}{{\rm{G}}}_{C}^{P}={{\rm{G}}}_{[\{{c}_{1},{c}_{2}\},p]}^{[\{L,R\},N]}=C\ast P={{\rm{c}}}^{{\rm{p}}}\frac{({{\rm{2}}}^{{\rm{N}}}+{{\rm{2}}}^{{\rm{N}}/{\rm{2}}})}{2},\quad N=even\\ {{\rm{G}}}_{C}^{P}={{\rm{G}}}_{[\{{c}_{1},{c}_{2}\},p]}^{[\{L,R\},N]}=C\ast P={{\rm{c}}}^{{\rm{p}}}\frac{({{\rm{2}}}^{{\rm{N}}/{\rm{2}}})}{2},\quad N=odd\end{array}$$

where (C) is the number of possible colour sequences, (P) denotes the unique generated patterns, (N) is the number of the motifs, (p) is the number of possible available positions on the motif, (c) is the number of colours (materials with different emissivity) and (G) denotes the unique number of generated functions.

We theoretically studied a plethora of different generated patterns; for some selected cases, we validated the results experimentally. Through the parametric coupled numerical models we identified the fundamental properties that govern the overall emissivity behaviour and manipulate the emissivity curve (Methods, Videos 6 and 7). Assuming a grey diffuse body, ε(T) = α(Τ), we calculated the effective thermal emissivity through the following relation, which is the ratio of the total amount of energy that leaves the patterned surface to that emitted from a black-body area and incorporates all the geometrical characteristics of the surface.

2 $${\varepsilon }_{eff}({\rm T})=\frac{Q}{A\sigma ({T}^{4}-{T}_{env}^{4})}$$

**Figure 4:**
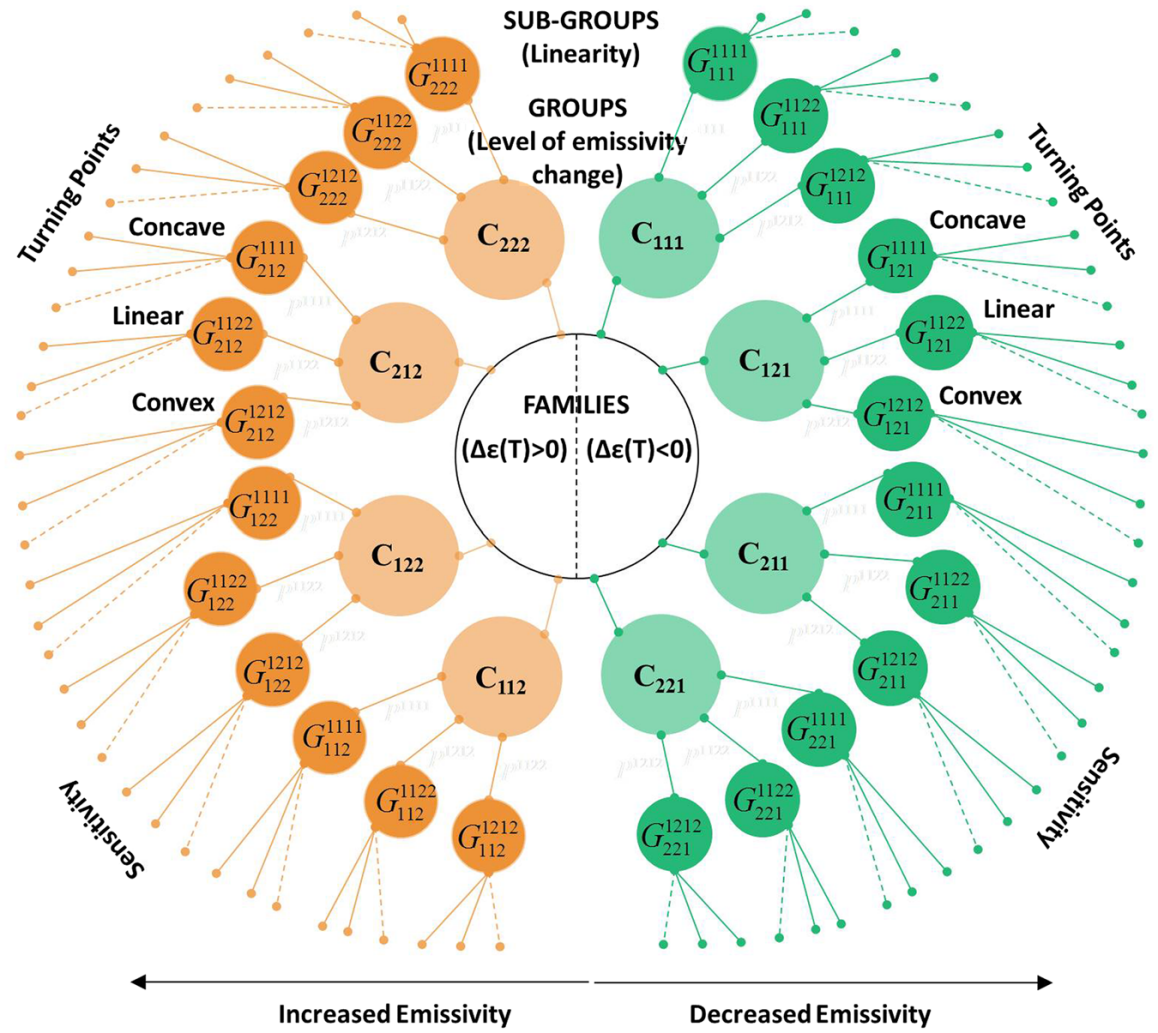
Generation and classification of emissivity functions according to: i. Two major families of equations are generated. Increased (Δε > 0) or decreased emissivity (Δε < 0) for closed-to-open motifs with different level of the emissivity change (Δε), (4 groups for each family), ii. Degree of linearity (10 subgroups for each group), and iii. Determination of sensitivity and turning, inflection points.

Entire families of different emissivity curves can be generated and classified into different levels through the combination of the orientation and the colours of the motifs, Fig. 4. Three main levels of classification exist according to: a) the change in the emissivity (negative Δε_eff_ < 0 or positive change Δε_eff_ > 0) and the level of change, b) the linearity of the path that the curve will follow to reach the min/max values of (ε_eff_), and c) the sensitivity of the material to temperature (Δε_eff_/ΔΤ).

### 1^st^ level of classification – Change in emissivity and degree of change

In the first-level, the classification of the ε_eff_(${{\rm{G}}}_{C}^{P}$,T) functions concerns the different colour sequences. In the 1^st^ family of equations, the emissivity increased (Δε_eff_ > 0), and in the 2^nd^ family of equations, the emissivity decreased (Δε_eff_ < 0) as a function of the temperature, Fig. 4.

For each family, four different groups exist, according the colour sequence C[{c_1_,c_2_},3] for a certain orientation sequence, where each group leads to a different degree of emissivity change. This is valid in the case where the motifs are transformed from the closed to the open state. In the case where the motifs transformed from the open to the closed state, the above statement is true only if we reverse the colour sequences.

**Figure 5:**
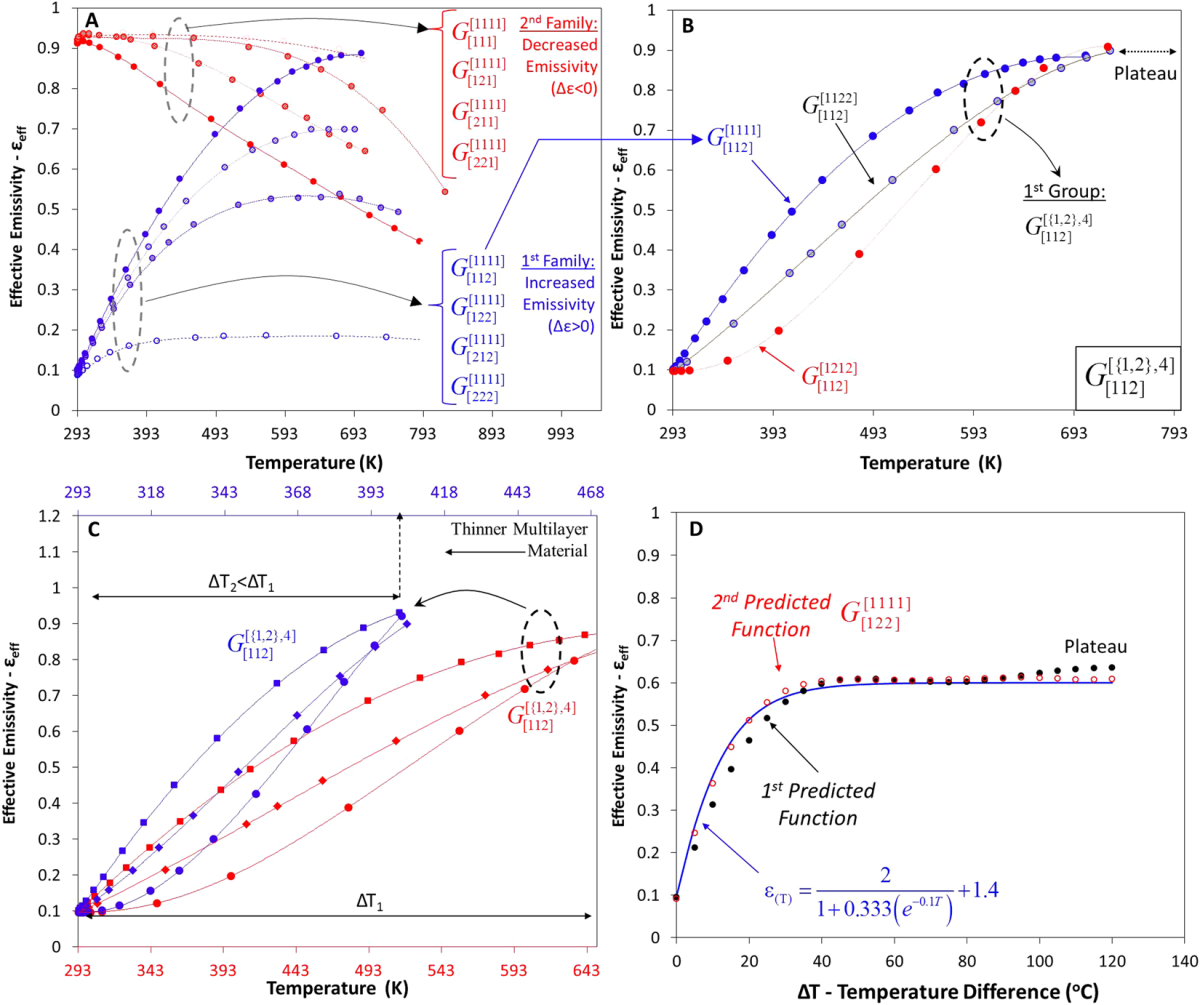
Generated effective emissivity functions of the finite-strip problem according to the 1^st^ and 2^nd^ classification. (**A**) Two generated families with four groups of functions for pattern ${{\rm{G}}}_{C}^{[1111]}$(1^st^ classification). (**B**) Generated subgroups of functions (2^nd^ classification) for the combinations ${{\rm{G}}}_{[112]}^{[1111]},{{\rm{G}}}_{[112]}^{[1122]},{{\rm{G}}}_{[112]}^{[1212]}$. Each group is consisted by 10 functions. (**C**) Transformation of the functions for a more sensitive multilayer film. (**D**) Predicted generated emissivity function ${{\rm{\varepsilon }}(G}_{[122]}^{[1111]},{\rm T})$ vs the set function (predetermined bounded function).

Figure 5a presents the generated emissivity functions of ${{\rm{\varepsilon }}(G}_{[\{{c}_{1},{c}_{2}\},3]}^{[1111]})$ for all colour sequences and for pattern P[1111] ≡ P[RRRR]. Each colour sequence leads to a different global max/min value for any generated pattern. By analysing the emissivity functions, we may observe that the colour sequences [{222},{212},{122},{112}] generate emissivity functions with positive change (Δε > 0) (1^st^ family of Fig. 4 and Fig. 5a), whereas the inverse colour sequences $\underset{\bar{2}=1}{\overset{\bar{1}=2}{\longrightarrow }}[\{{\rm{111}}\},\{{\rm{121}}\},\{{\rm{211}}\},\{{\rm{221}}\}]$ generate emissivity functions with a negative emissivity change (Δε < 0) (2^nd^ family of Fig. 4 and Fig. 5a).

The colour sequence of the 1^st^ family, which consists of only low-emissivity materials {222}, increases the emissivity ${{\rm{\varepsilon }}(G}_{[222]}^{[1111]},{\rm T})$ of the surface because the view factor of the surface changes. The entire smart surface is composed of a material of low emissivity (internal and external surfaces of the motifs); during the opening of the motifs, the interaction of their geometry slightly increases the effective emissivity of the surface. This is logical because the flat surface is transformed to a patterned surface. Similar changes can be observed in surfaces that have formed open cylindrical cavities^10^.

In the case where two different colours have been applied on the motifs with colour sequences [{212},{122},{112}], the emissivity change is positive and considerably higher. When the motifs are closed, colour sequence {112} has a low-emissivity material on the outer surface {2}, whereas the internal surfaces have a high-emissivity material {11}. During the transformation of the motifs, the internal high-emissivity materials are exposed to the environment, and their emissivity value dominates. The surface increases their ability to radiate energy as the motifs open wider and wider. In addition, the view factor of the surface alters the ability of the surface to radiate the energy; however, its influence is smaller.

On curves ${{\rm{G}}}_{[212]}^{[1111]}$ and ${{\rm{G}}}_{[122]}^{[1111]}$, which have a {212} colour sequence and a {122} colour sequence, respectively, we can observe a small decrease in the emissivity at higher temperatures (after the global maxima of each curve). This behaviour is observed because at this temperature, each motif shades the neighbouring motif.

### 2^nd^ level of classification – Linearity, convexity

The secondary-level classification is related to the orientation permutations/sequences of the motifs, and determines the linearity of the path that the curve will follow to reach the min/max values of (ε_eff_); each group includes ten functions with different measure of linearity, until the max/min value of the function is reached. Therefore, concave ${{\rm{\varepsilon }}(G}_{[112]}^{[1111]})$, convex $\varepsilon ({{\rm{G}}}_{[112]}^{[1212]})$, or ‘linear’ $\varepsilon ({{\rm{G}}}_{[112]}^{[1122]})$ functions are generated (Fig. 5b) for all colour sequences [{c_1_,c_2_},3]. All generated functions of each group are restricted within an upper limit ${{\rm{\varepsilon }}(G}_{[112]}^{[1111]})$ and a lower limit ${{\rm{\varepsilon }}(G}_{[112]}^{[1212]})$; the emissivity of all other patterns is restricted within these limits (Methods). The measure of linearity was correlated with the shape of the curve^44^, and could be characterised and categorised using different mathematical quantities, such as the ellipticity, eccentricity or rectangularity. To compare the linearity of the generated functions of each group, we employed ellipticity measures.

By measuring the ellipticity of a finite set of points, it is possible to classify our curves. The central moment *μ*_*pq*_ of the (pq) order is the following.

3 $${\mu }_{pq}=\frac{1}{S}\sum {(T-{T}_{c})}^{P}{(\varepsilon -{\varepsilon }_{c})}^{q}$$

4 $$(\bar{{\rm T}},\bar{\varepsilon })=(\frac{1}{S}\sum {{\rm T}}_{i},\frac{1}{S}\sum {\varepsilon }_{i})$$

Here, $(\bar{{\rm T}},\bar{\varepsilon })$ is the average value of each coordinate (Τ_i_, ε_i_). The linearity can be correlated with the ellipticity of the points of the generated function, and can be expressed as in the following.

5 $$\begin{array}{c}a=\sqrt{2[{\mu }_{20}+{\mu }_{02}+\sqrt{{({\mu }_{20}-{\mu }_{02})}^{2}+4{\mu }_{11}^{2}}]/{\mu }_{00}}\\ b=\sqrt{2[{\mu }_{20}+{\mu }_{02}-\sqrt{{({\mu }_{20}-{\mu }_{02})}^{2}+4{\mu }_{11}^{2}}]/{\mu }_{00}}\\ \lambda =1-a/b,\quad 0\le \lambda \le 1\end{array}$$

The linear curves are denoted as λ ≈ 1, whereas the non-linear yields values of λ < 1. The value of the linearity measure of the 1^st^ curve $\varepsilon ({{\rm{G}}}_{[112]}^{[1111]},T)$ is λ = 0.893, of the 2^nd^ curve $\varepsilon ({{\rm{G}}}_{[112]}^{[1122]},T)$ is λ = 0.97, and of the 3^rd^ $\varepsilon ({{\rm{G}}}_{[112]}^{[1212]},T)$ is λ = 0.91. Pattern ${{\rm{G}}}_{[112]}^{[1122]}$ generates functions which are almost linear (Fig. 5b,c) prior to the function reaching a plateau.

Other, simpler tools are related to the identification of the convexity of the generated function. The convexity of the discrete function ${{\rm{\varepsilon }}(G}_{C}^{P},T)$ between three points can be expressed using the following simple inequality.

6 $$\varepsilon (i-1)+\varepsilon (i+1)-2\varepsilon (i) > 0$$

We may observe that pattern P[1111], which is the upper limit of the group, Fig. 5b, always leads to $\varepsilon ({{\rm{G}}}_{[112]}^{[1111]},T)$ emissivity functions that are concave, and which present strong non-linear behaviour. On the other hand, pattern [1212], which presents the lowest limit of the group, Fig. 5b, always leads to $\varepsilon ({{\rm{G}}}_{[112]}^{[1212]},T)$ emissivity functions that are convex, and which present also strong non-linear behaviour. In contrast, pattern [1122] always leads to emissivity functions $\varepsilon ({{\rm{G}}}_{[112]}^{[1122]},T)$ that are almost linear, until the emissivity value reaches its global maximum, Fig. 5b. The different behaviour of the emissivity is related to the colours of each motif, the interaction with the colours of the neighbour motifs (orientation of the motifs), and the geometry of the motifs. Essentially, in pattern P[1111], a cavity with high-emissivity materials interacts with a flat surface with low-emissivity material. On the other hand, in pattern [1212], a cavity with high-emissivity materials interacts with another cavity with high-emissivity materials (Methods). The view factor and the combination of the interacting colours determine the radiative heat flux and the effective emissivity of the material. The degree of influence of the view factor cannot be isolated. Moreover, we can observe that there is a region where all functions reach a global maximum. In this temperature region, all motifs have undergone the same transformation, and the sequence of the colours leads to approximately the same emissivity value.

### 3^rd^ level of classification – Sensitivity and similarity

Other parameters which affect the overall radiative behaviour of the patterned surface are related to i) the physical properties of the materials, ii) the shape and iii) the distance between the motifs, and iv) thickness of the multilayer material. In the case where the thickness of the multilayer material decreases, the emissivity function maintains its behaviour; however, with a greater sensitivity (Fig. 5c). It was found that all generated functions adapt their form to achieve the min/max values within a smaller temperature span; however, their dimensionless form has extremely similar characteristics. The value of the linearity measure for the two different thicknesses (H_1_ > H_2_) of the 1^st^ curve $\varepsilon ({{\rm{G}}}_{[112]}^{[1111]},T)$ ranges within 0.893 < λ < 0.930, of the 2^nd^ curve $\varepsilon ({{\rm{G}}}_{[112]}^{[1122]},T)$ ranges within 0.97 < λ < 0.99, and of the 3^rd^ $\varepsilon ({{\rm{G}}}_{[112]}^{[1212]},T)$ ranges within 0.91 < λ < 0.930 (Table 1).

**Table 1:** Linearity and similarity measures. Linearity of the curves for different multilayer thickness and similarity between these curves.

		$\varepsilon ({{\bf{G}}}_{[{\bf{112}}]}^{[{\bf{1111}}]},{\boldsymbol{T}})$		$\varepsilon ({{\bf{G}}}_{[{\bf{112}}]}^{[{\bf{1122}}]},{\boldsymbol{T}})$		$\varepsilon ({{\bf{G}}}_{[{\bf{112}}]}^{[{\bf{1212}}]},{\boldsymbol{T}})$	
		*H*_1_	*H*_2_	*H*_1_	*H*_2_	*H*_1_	*H*_2_
**Convexity**		Concave Nonlinear <1		Linear ≈ 1		Convex Non-linear <1	
**Linearity** 0 ≤ λ ≤ 1	λ_1_	**0.893**	**0.930**	**0.970**	**0.99**	**0.930**	**0.910**
**Similarity** 1 ≤ s ≤ 1	s_1_	**0.996**		**0.998**		**0.999**	
	s_2_	**0.999**		**0.999**		**0.999**	

Moreover, we used simple schemes to validate the similarity between the shapes of the curves for the two different thicknesses, such as the normalised correlation and Pearson’s correlation coefficient. Their values indicated the similarity of the two generated curves with different thickness for the same colour and orientation sequences. The measure of similarity receives values between −1 ≤ s ≤ 1; for s ≈ 1, the correlation/similarity is excellent.

7 $${s}_{1}=\frac{(i\sum {\varepsilon }_{C}^{P}{(i)}_{1}{\varepsilon }_{C}^{P}{(i)}_{2}-\sum {\varepsilon }_{C}^{P}{(i)}_{1}\sum {\varepsilon }_{C}^{P}{(i)}_{2})}{\sqrt{i\sum {({\varepsilon }_{C}^{P}{(i)}_{1})}^{2}-{(\sum {\varepsilon }_{C}^{P}{(i)}_{1})}^{2}}\sqrt{i\sum {({\varepsilon }_{C}^{P}{(i)}_{2})}^{2}-{(\sum {\varepsilon }_{C}^{P}{(i)}_{2})}^{2}}}$$

8 $${s}_{2}=\frac{\sum {\varepsilon }_{C}^{P}{(i)}_{1}{\varepsilon }_{C}^{P}{(i)}_{2}}{\sqrt{{\sum ({\varepsilon }_{C}^{P}{(i)}_{1})}^{2}{\sum ({\varepsilon }_{C}^{P}{(i)}_{2})}^{2}}}$$

Here, ε_1_(i) and ε_2_(i) represent two emissivity curves which have been generated by the smart patterns of different thickness. The similarity between the two emissivity curves for three different patterns are: s = 0.996 for $\varepsilon ({{\rm{G}}}_{[112]}^{[1111]},T)$, s = 0.998 for $\varepsilon ({{\rm{G}}}_{[112]}^{[1122]},T)$, and s = 0.999 for $\varepsilon ({{\rm{G}}}_{[112]}^{[1212]},T)$ (Table 1). The emissivity curves maintain their characteristics; they only became ‘distorted’, and were located within a smaller ΔΤ (Fig. 5c). By calculating the correlation coefficients of three different functions—$$\varepsilon ({{\rm{G}}}_{112}^{1212}),\begin{array}{cc} & \varepsilon ({{\rm{G}}}_{112}^{1122}),\end{array}\begin{array}{cc} & \varepsilon ({{\rm{G}}}_{112}^{1111})\end{array}$$—versus the thickness, we concluded that the similarity in all cases ranged within 99.6–99.9%. Therefore, we may deduce that only two parameters determine the main characteristics of the curve, namely the orientation patterns and the colour sequences.

In a similar manner, if the motif has a fully deformable region, the behaviour remains the same; this region affects only the sensitivity of the curve (Δε_max_/ΔT) and may slightly modify the global min/max values (Methods), i.e. the emissivity of pattern $\varepsilon ({{\rm{G}}}_{[112]}^{[1111]})$ of the fully deformable motifs changes from 0.095 to 0.93 within ΔΤ = 152 °C; this results in a sensitivity of (Δε_max_/ΔT = 0.0055 °C^−1^). The emissivity of the partially deformable motifs changes from 0.095 to 0.93 within ΔΤ = 400 °C resulting to a sensitivity (Δε_max_/ΔT = 0.00208 °C^−1^) (Methods). Observing the convexity measure of the emissivity function, we conclude that the two curves are convex and present strong non-linear characteristics. The interactions between the colours determine the convexity of the emissivity curves and overcome the influence of the geometrical transformation of the motifs (fully and partially deformable motifs). Essentially, the interactions between the fully deformable motifs and the interactions between the partially deformable motifs lead to the same type of curves.

### Approximating a predetermined curve

In order to prove the efficiency of the design through this combinatorial design we selected a convex bounded equation ε(Τ) of certain characteristics with the purpose of predicting the sequences of colours and orientations that would yield the selected equation. We set a predetermined bounded equation ε(Τ) of the form $\varepsilon ({\rm T})=\frac{{a}_{1}}{{b}_{1}+{b}_{2}({e}^{-c{T}^{d}})}+{a}_{2}$, which can be configured to produce linear, concave or convex paths. Power (d) regulates the linearity of the equation; a_1_, a_2_, b_1_, and b_2_ regulate the emissivity change (Δε) with ΔΤ. By setting the lower and upper limits to ε_min_ = 0.1 and ε_max_ = 0.6, respectively, within the temperature change of ΔΤ = 80 °C, the following equation was obtained: *ε*(*T*) = (2)/(1 + 0.333(*e* ^−0.1*T*^)) + 1.4. After examining the sequences of colours and orientations, we concluded that $\varepsilon ({{\rm{G}}}_{[122]}^{[1111]},T)$ best approximates the aforementioned equation. These specific sequence produced a curve whose shape was 99.89% similar (Eq. 7) to that of the predetermined/set equation, and with the same measure of linearity (λ_set_ = 0.86, λ_gener_ = 0.85) (Eq. 5) and emissivity change (Δε_set_ = 0.5, Δε_gener_ = 0.517) (Fig. 5d). Essentially, the set curve (black line) can be generated (red dots) if we use a pattern with a P[RRRR] ≡ P[1111] orientation sequence, a high-emissivity material in Position 1, and a low-emissivity material on Positions 2 and 3, C[122].

## Experimental Verification of the Effective Emissivity of Different Generated Curves

**Figure 6:**
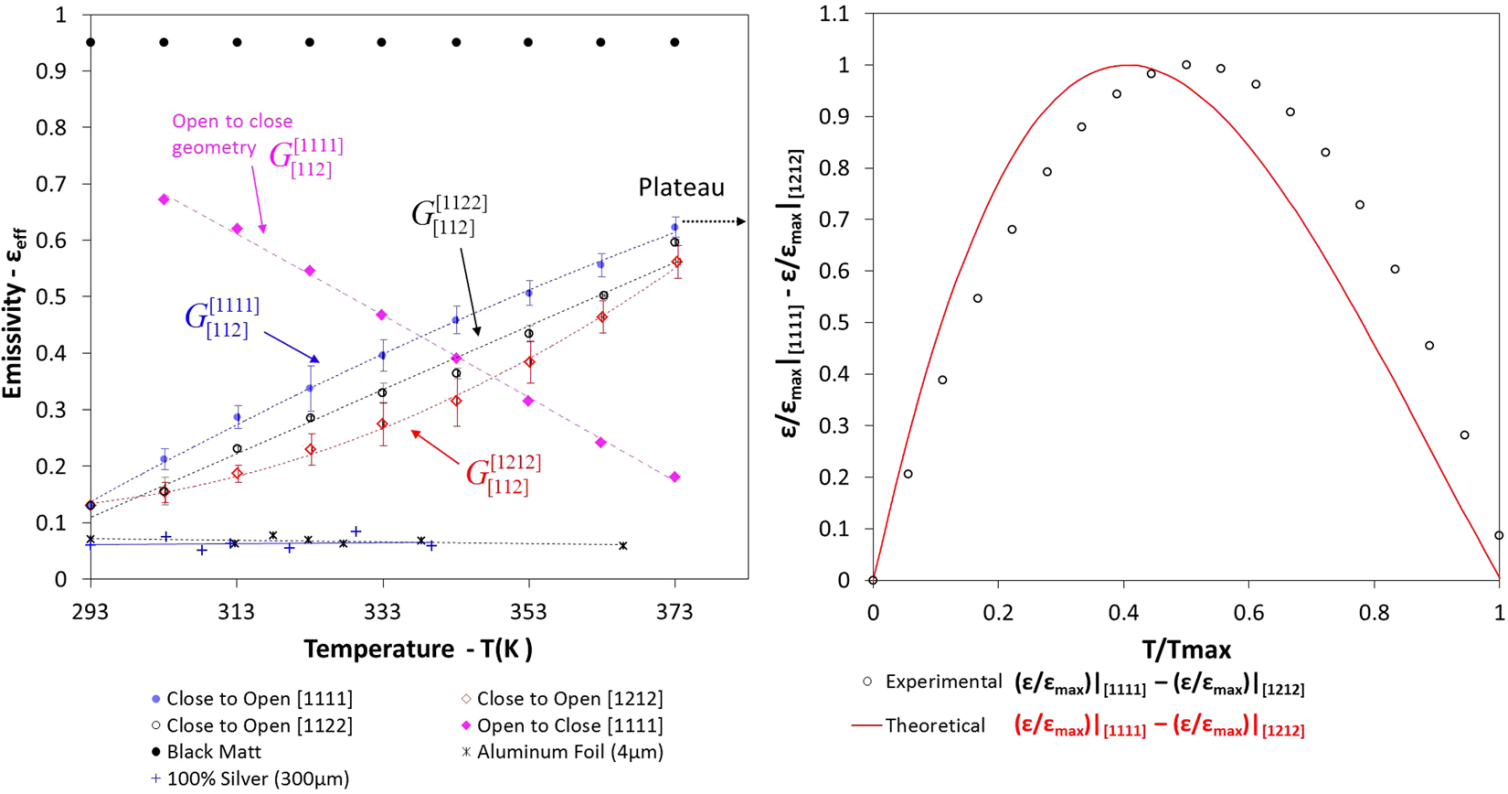
(**A**) Experimentally generated effective emissivity functions with different linearity measure of the finite-strip problem for the following close to open patterns; ${{\rm{G}}}_{[112]}^{[1111]},{{\rm{G}}}_{[112]}^{[1122]},{{\rm{G}}}_{[112]}^{[1212]}$ and open to close pattern ${{\rm{G}}}_{[112]}^{[1111]}$. (**B**) Comparison of the experimental and theoretical curves of the dimensionless emissivity difference between the upper and lower limits ${\rm{\varepsilon }}({{\rm{G}}}_{[112]}^{[1111]},{\rm T})-{\rm{\varepsilon }}({{\rm{G}}}_{[112]}^{[1212]},{\rm T})$ for patterns P[1111] and P[1212]. The trend of the curves between the theoretically generated functions and the acquired experimental functions is almost identical.

Finally, we experimentally verified the theoretical results through a plethora of measurements, using calorimetric techniques. We selected three particular patterns $\varepsilon ({{\rm{G}}}_{[112]}^{[1111]}),\varepsilon ({{\rm{G}}}_{[112]}^{[1122]}),\varepsilon ({{\rm{G}}}_{[112]}^{[1212]})$ because they have different measures of linearity, and they define the extreme lower and higher limits of the possible generated thermal emissivity functions (Fig. 6a). All selected surfaces have the same colour sequence, C[112]. On the internal surfaces of the motifs, namely the 1^st^ and 2^nd^ position, the emissivity is high (ε_1_ ≈ 0.95 ≡ c_1_), and on the external surface of the motif, namely the 3^rd^ position, the emissivity is low (ε_2_ ≈ 0.1 ≡ c_2_). The orientation of the motifs on the three tested surfaces are P[1111], P[1122], and P[1212], respectively.

The change in emissivity is approximately Δε ≈ 0.47 for all curves. The experimental results generated three curves of different measures of linearity, which is in agreement with our prediction within the temperature span of ΔΤ ≈ 80 °C. Small differentiations in the behaviour of the acquired and the theoretical curves may be present owing to the specular nature of the aluminium surface, as well as the inequality of the absorptivity and the emissivity of the external aluminium surface, α_al_ > ε_al_. Moreover, small non-homogeneous regions in the multilayer material may lead to small deviations regarding the deformation of the motif.

The 1^st^ pattern $\varepsilon ({{\rm{G}}}_{[112]}^{[1111]})$ leads to a concave function (blue circles), whereas the 3^rd^ pattern $\varepsilon ({{\rm{G}}}_{[112]}^{[1212]})$ leads to a convex function (red diamonds), (Fig. 6a). The 2^nd^ pattern $\varepsilon ({{\rm{G}}}_{[112]}^{[1122]})$ leads to an almost linear function (black circles). Moreover, a P[1111] pattern with the same colour sequence {112} was tested, using the opposite transformation of the motifs (open to close state, pink diamonds). The experimentally studied patterned surfaces during the heating and cooling stage are presented also in Fig. S2 and Video S5.

The solid black dots represent the emissivity of the high-emissivity coating, whereas the blue crosses and black asterisks represent the emissivity of aluminium and of a 100% silver foil, respectively. The emissivity measurements of different materials were obtained in order to develop benchmark experiments, and to ensure that the apparatus and the measurement methodology are accurate. The exact experimental methodology is described in detail in the Supplementary Information.

The experimental measurements are extremely laborious and sensitive due to the complexity of the patterned surfaces and the measurements at low temperature levels. To compare the theoretically generated and the acquired experimental functions, we compared the dimensionless difference of the upper $\varepsilon ({{\rm{G}}}_{[112]}^{[1111]})$ and lower functions $\varepsilon ({{\rm{G}}}_{[112]}^{[1212]})$. The difference between these two functions vs the dimensionless temperature provides a qualitative comparison of their linearity measure. The trend of the curves between the theoretical generated functions and the acquired experimental functions is almost identical, Fig. 6b. A small shift appears because of the aforementioned reasons. Observing the emissivity difference between these two patterns, namely $(\varepsilon ({{\rm{G}}}_{[112]}^{[1111]})-\varepsilon ({{\rm{G}}}_{[112]}^{[1212]}))$ vs the dimensionless temperature $(T/{T}_{\max })$, we may conclude that the theoretical results are in very good agreement with the experimental measurements, Fig. 6b.

## Conclusions

By identifying and handling these fundamental properties—orientation and colour sequences—we designed the thermal emissivity function of a patterned surface. The classification of the generated curves and the similarities owing to the existence of invariant properties limit the number of combinations that need to be considered. We drastically altered the emissivity value (approximately Δε ≈ 0.47) within the temperature span of ΔΤ ≈ 80 °C, Fig. 6 and ΔΤ ≈ 37 °C, Fig. S4; thus, we developed integrated, low-weight, cost-effective, and programmable thermal-management materials/surfaces.

Using these materials and through the combinatorial design strategy, we may design any bounded function within certain interval. Of course, it is not possible to generate emissivity values larger than unit and smaller than zero (0 < ε < 1), (practically 0.03 ≤ ε ≤ 0.96). Theoretically, we can approximately design any strictly monotonic or non-monotonic function with one turning and one inflection point (i.e. theoretically we could predict all the characteristics of the material to approximately design a trigonometric function, ε(T) = sin(T) but within certain intervals, namely [0 < T ≤ π/2] or [0 < T < 2π/3]; however, we cannot return to the initial emissivity value). Τhis is logical because the emissivity must receive a global minimum or maximum value, as well as a steady value ε > ε_min_, beyond a temperature level (T). In addition, a steady value for T > T_max_ is achievable; practically, however, the maximum temperature is determined by the physical properties of the materials. More complex functions may be designed with limitations (particular intervals). Hence, in our future investigation, we will focus on the design of more complex functions, implementing more complex motifs (with more degrees of freedom) with or without nonlinear material properties.

These smart patterned surfaces present the following advantages compared with the aforementioned materials/ΜΕΜS/devices. A) Their most important advantage is that we may use these fundamental properties to design the entire emissivity vs temperature curve; currently, there is no study or technological achievement in which the aforementioned advantages have been introduced. B) These smart patterned surfaces function passively; this means that they use energy from their environment, as opposed to MEMs, which use high-voltage power supplies in order to change the effective thermal emissivity. C) They have the ability to change their emissivity to a great extent; the change may either be positive or negative (Δε < 0 or Δε > 0), as opposed to certain materials for which their emissivity decreases as the temperature increases. D) The material can be developed to passively react over a very broad range of thermal requirements (i.e. −270 °C to +350 °C). E) They are low-weight compared with various other devices that are considerably efficient; however, these devices have a limited range of application and are very heavy.

Radiative thermal management is crucial for every application subsystem on Earth and in Space where in the latter, thermal radiation is the only heat transfer mechanism. The present work can significantly contribute to the future thermal design of various energy systems— such as buildings and satellites/spacecraft for Space exploration—and sensors for the directional identification of a heat source or for handling different wavelengths. The potential applications are numerous. More specifically, in Space applications, all approaches mentioned in the introduction suffer from major drawbacks: the devices are extremely complex and heavyweight; MEMs are extremely complex, high-cost and low-performing; certain materials require power supplies and are low-performing or incapable of activation at various temperature ranges. These smart materials may change their emissivity from 0.03 to 0.9, as well as their absorptivity for small temperature differentials (ΔΤ < 20 °C) at any temperature level. Meanwhile, we can predetermine the behaviour of the effective thermo-optical properties as a function of temperature. In addition, practically, we can design these materials to resist in UV radiation or to absorb particular wavelengths. Using these low-cost materials, we can passively control the temperature of the systems and sub-systems of a satellite through the regulation of the absorptivity/emissivity ratio, and we may reduce the weight and the complexity of the overall system. Furthermore, this work may lead to the development of “4D materials” and thermal adaptable materials.

## Methods

### Theoretical considerations of the combinatorial strategy

The generated functions $\varepsilon ({{\rm{G}}}_{C}^{P},T)=\varepsilon ({{\rm{G}}}_{[\{{c}_{1},{c}_{2}\},p]}^{[\{R,L\},N]},T)\underset{{c}_{1}\equiv 1,{c}_{2}\equiv 2}{\overset{R\equiv 1,L\equiv 2}{\longrightarrow }}\varepsilon ({{\rm{G}}}_{[\{1,2\},p]}^{[\{1,2\},N]},T)$ of a mono-translational or ditranslational lattice can be expressed as the combination of two finite ordered lists of elements. Regarding the strip problem, the combination of two finite ordered lists of elements are P[{R,L},N] and C[{c_1_,c_2_},p]. A motif with 1-fold rotational symmetry-r1 on a unit cell with a 2-fold rotational symmetry-r2 generates two different orientations {R,L}. For N = 4 motifs, the generated sequences are the following: [{RRRR}, {RRRL}, {RLRR}, {LRRR}, {RLRL},…,{LLLL}], (Table 2). In a similar manner, the colour sequences for two colours in three positions (p) are [{c_1_,c_1,_c_1_}, {c_1_,c_1,_c_2_}, {c_1_,c_2,_c_1_},…,{c_2_,c_2,_c_2_}]. All mirror patterns lead to equivalent emissivity values e.g. the sequence $\{[\mathrm{RLRR}]\equiv [\mathrm{LRLL}]\underset{L\equiv 2}{\overset{R\equiv 1}{\longrightarrow }}[\mathrm{1211}]\equiv [\mathrm{2122}]\}$, and generate exactly the same emissivity function $\varepsilon ({{\rm{G}}}_{C}^{[1211]},T)=\varepsilon ({{\rm{G}}}_{C}^{[2122]},T)$. A first screening excludes all mirror [1211] ≡ [2122] or self-similar patterns [1122] ≡ [1122], thus reducing the number of patterns which lead to unique solutions. As previously described, two different cases are presented in the mono-translational lattice: N = odd number and N = even number. If (N) is an odd number, 2^N^ patterns can be generated; therefore, ${{\rm{2}}}^{N}/2$ are the mirror patterns and ${{\rm{2}}}^{N}/2$ are the unique generated patterns.

**Table 2:** Generated Sequences as a Function of the Number of Motifs on a Strip. Comparison between the generated unique and mirror patterns for odd and even N. The blue colour represents all self-similar patterns.

N = *Number of Motifs*									
		**N** = **1**		**N = 2**		**N = 3**		**N = 4**	
		Pattern	Mirror Pattern	Pattern	Mirror Pattern	Pattern	Mirror Pattern	Pattern	Mirror Pattern
**Sequences**	1	R	L	RR	LL	RRR	LLL	RRRR	LLLL
	2			RL	RL	RRL	RLL	RRRL	RLLL
	3			LR	LR	RLR	LRL	RLRL	RLRL
	4					LRR	LLR	LRRR	LLLR
	5							LRRL	RLLR
	6							LRLR	LRLR
	7							RRLR	LRLL
	8							RLRR	LLRL
	9							LLRR	LLRR
	10							RRLL	RRLL

For N = even number, the generated mirror patterns are ≠ ${{\rm{2}}}^{N}/2$, and the unique generated patterns are ≠ ${{\rm{2}}}^{N}/2$. This is valid owing to the existence of the self-similar patterns (2^N/2^). As a consequence, the unique generated patterns are equal to the total generated patterns minus the unique mirror patterns, P = 2^N^ − (2^N^ − 2^N/2^)/2 = (2^N^ + 2^N/2^)/2, or to the sum of the unique generated patterns and the self-similar mirror patterns, P = (2^N^ − 2^N/2^)/2 + 2^N/2^ = (2^N^ + 2^N/2^)/2.

**Figure 7:**
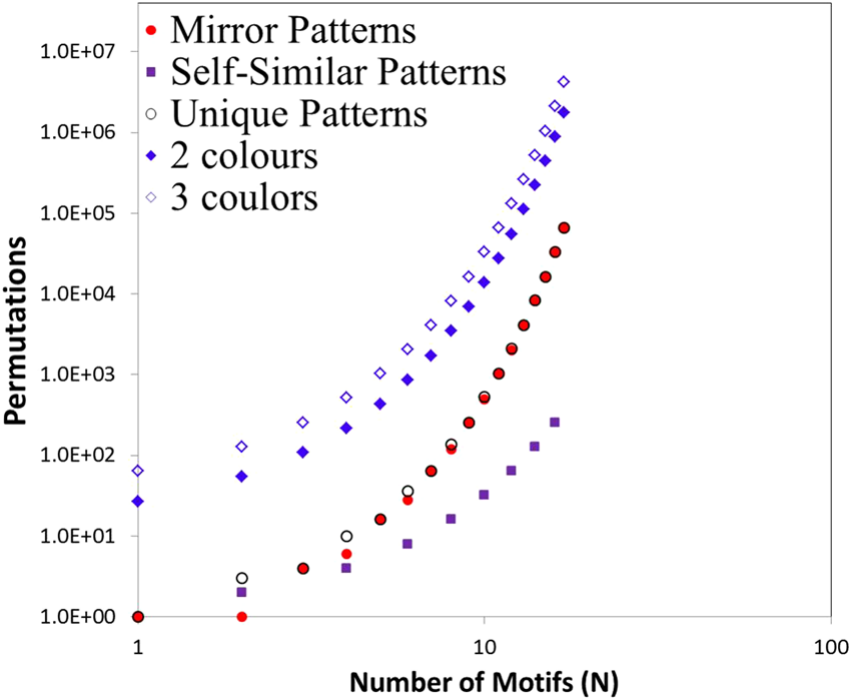
Generated combinations of unique colour–orientation patterns as a function of the number of motifs on a finite strip.

In the case of one colour, all the sequences generate approximately the same curve. In contrast, the combination of at least two different colours generates an entire family of different emissivity curves. A strip with 4 motifs (positions) has 10 unique patterns, and the use of 2 different colours in 3 possible positions generates 80 unique sequences. In the Table 3 and Fig. 7, we list all unique generated functions for N ≤ 7 for 1 colour, 2 colours, and 3 colours. It is important to be mentioned that the exact integer sequences of the unique mirror patterns and the unique number of the generated patterns ∀*N* may be found in the On-Line Encyclopedia of Integer Sequences (A007179 & A051437) in two studies of other scientific disciplines^45,46^.

**Table 3:** Possible Rotational (orientation) and Colour Permutations. Calculation of the unique patterns and functions for N = 1 to N = 7 motifs with 1-fold rotational symmetry, 1–3 colours, and p = 3 positions on the motifs.

Motifs (N)	Possible Patterns [{L,R},N]	Mirror Patterns (P_M_)	Self-similar Patterns (P_S_)	Unique Sequences (P)	Unique Functions for ${{\bf{G}}}_{{\boldsymbol{[}}{\boldsymbol{\{}}{{\bf{c}}}_{{\bf{1}}}{\boldsymbol{\}}}{\boldsymbol{,}}{\bf{p}}{\boldsymbol{]}}}^{{\boldsymbol{[}}{\boldsymbol{\{}}{\bf{1}}{\boldsymbol{,}}{\bf{2}}{\boldsymbol{\}}}{\boldsymbol{,}}{\bf{N}}{\boldsymbol{]}}}$	Unique Functions for ${{\bf{G}}}_{{\boldsymbol{[}}{\boldsymbol{\{}}{{\bf{c}}}_{{\bf{1}}}{\boldsymbol{,}}{{\bf{c}}}_{{\bf{2}}}{\boldsymbol{\}}}{\boldsymbol{,}}{\bf{p}}{\boldsymbol{]}}}^{{\boldsymbol{[}}{\boldsymbol{\{}}{\bf{1}}{\boldsymbol{,}}{\bf{2}}{\boldsymbol{\}}}{\boldsymbol{,}}{\bf{N}}{\boldsymbol{]}}}$	Unique Functions For ${{\bf{G}}}_{{\boldsymbol{[}}{\boldsymbol{\{}}{{\bf{c}}}_{{\bf{1}}}{\boldsymbol{,}}{{\bf{c}}}_{{\bf{2}}}{\boldsymbol{,}}{{\bf{c}}}_{{\bf{3}}}{\boldsymbol{\}}}{\boldsymbol{,}}{\bf{p}}{\boldsymbol{]}}}^{{\boldsymbol{[}}{\boldsymbol{\{}}{\bf{1}}{\boldsymbol{,}}{\bf{2}}{\boldsymbol{\}}}{\boldsymbol{,}}{\bf{N}}{\boldsymbol{]}}}$
**Even**	2 ^N^	(2^N^−2^N/2^)/2	2 ^N/2^	(2 ^N^ + 2 ^N/2^)/2	c^p^ (2 ^N^ + 2 ^N/2^)/2		
**Odd**		2 ^N^/2	0	2 ^N^/2	c^p^ (2 ^N^)/2		
1	2	1	0	1	1	8	64
2	4	1	2	3	3	24	128
3	8	4	0	4	4	32	256
4	16	6	4	10	10	80	512
5	32	16	0	16	16	128	1024
6	64	28	8	36	36	288	2048
7	128	64	0	64	64	512	4096

### Parametric numerical modelling

All possible unique sequences for N = 4 motifs were solved through steady-state or transient coupled thermo-mechanical models for different temperature levels using the COMSOL Multiphysics. The physical properties, as well as the dimensions, are presented in detail (See Supplementary Information). These models generate all the unique discrete emissivity functions versus temperature. In the case of large displacements, non-linear phenomena appear. The non-linear behaviour originates from the geometrical non-linearity that is due to the small thickness and the large displacements and rotations of the multilayer material, and not from the properties of the material itself^42^. For this reason, the strain tensor with the non-linear terms should be considered. In our case, it is necessary to solve a coupled thermo-mechanical and geometrically non-linear problem which incorporates the interactions of the body caused by thermal radiation as well. The Green–Lagrange strain tensor represents the strains, and the Second Piola–Kirchoff stress tensor represents the stresses. We used structured quadrilateral elements to model the overall phenomenon.

9 $${e}_{el}=\frac{1}{2}[{(\nabla u)}^{T}+\nabla u+{(\nabla u)}^{T}\nabla u]-a(T-{T}_{ref})$$

Owing to the complexity of the problem, we developed patterned surfaces which can be modelled through 2D plain strain problems (Fig. 3). We solved these models parametrically in order to study the interactions between the motifs and to predict their final geometry, the temperature field, and the effective emissivity of the patterned surfaces. All the unique orientation and colour sequences were solved and classified accordingly (Fig. 4). The parameters of interest are the radiosity, emissivity, temperature, and the irradiation. The radiosity leaving a surface is defined by

10 $${J}_{i}={\rho }_{i}{G}_{i}+{\varepsilon }_{i}\sigma {T}_{i}^{4}$$

Here, (*ρ*_*i*_) and (*ε*_*i*_) are the surface reflectivity and emissivity, respectively, and (i) denotes the surface of the low- or the high-emissivity material. In general, the irradiation, (G), of the surface can be written as a sum, as in the following.

11 $${G}_{i}={G}_{m,i}+{F}_{a,i}\sigma {T}_{a}^{4}$$

Here, (*G*_*m*_) is the mutual irradiation from other boundaries, (*F*_*a*_) is an ambient view factor, and (*T*_*a*_) is the assumed far-away temperature in the directions included in (*F*_*a*_). In fact, (*G*_*m*_) is the integral over all visible points of a differential view factor (F) multiplied by the radiosity (J) of the corresponding source point. In the discrete model, (*G*_*m*_) may be expressed as the product of a view factor matrix and a radiosity vector.

12 $${J}_{i}={\rho }_{i}[{G}_{m,i}(J)+{F}_{a,i}\sigma {T}_{a}^{4}]+{\varepsilon }_{i}\sigma {T}_{i}^{4}$$

Assuming an ideal grey body, Eq. 12 becomes

13 $${J}_{i}=(1-{\varepsilon }_{i})[{G}_{m,i}(J)+{F}_{a,i}\sigma {T}_{a}^{4}]+{\varepsilon }_{i}\sigma {T}_{i}^{4},\quad i={\rm{1}},{\rm{2}},\ldots ,k.$$

Equation (12) results in an equation system in (*J)*, which is solved in parallel with the temperature equation, (*T)*; (k) expresses the number of surfaces which the overall structure consists of. We predicted the geometric transformation of the motifs and the change in the ambient view factor during their interaction for two different cases—partially and fully deformable area—by solving the transient problem (Videos S6 and S7).

**Figure 8:**
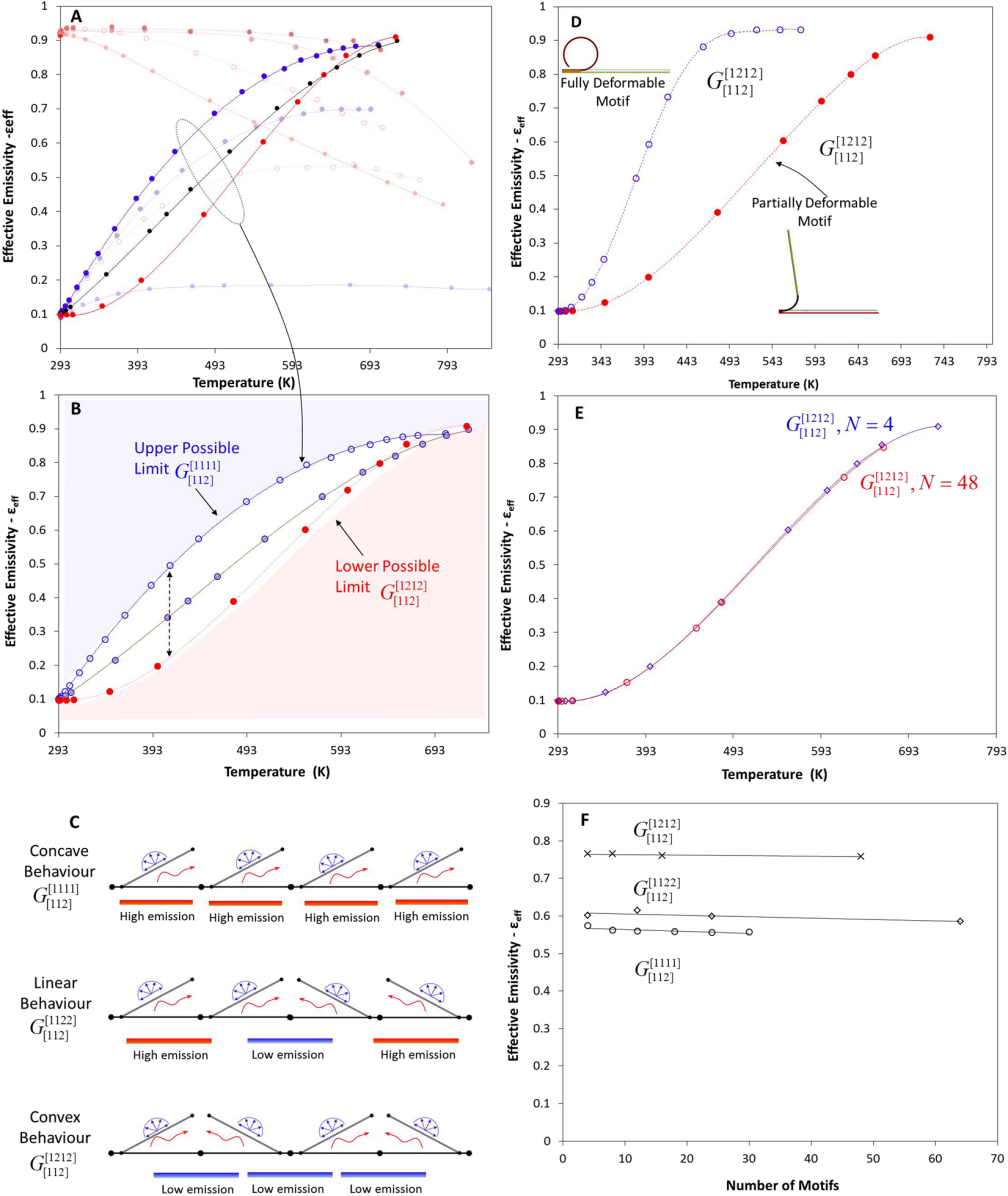
(**A**) Generated groups for P[1111] and for all colour sequences, ${{\rm{G}}}_{[\{{c}_{1},{c}_{2}\},3]}^{[1111]}$. (**B**) All orientation sequences P[{1,2},N] generate functions of different linearity measure, whereas the extreme sequences ${{\rm{G}}}_{[\{{c}_{1},{c}_{2}\},3]}^{[1111]}$, ${{\rm{G}}}_{[\{{c}_{1},{c}_{2}\},3]}^{[1212]}$ dictate the upper and the lower limit. (**C**) Schematic representation of the motifs’ interaction for three selected cases. (**D**) Comparison between the generated functions for a partial and a fully deformed motif for pattern ${{\rm{G}}}_{[\{{c}_{1},{c}_{2}\},3]}^{[1212]}$. If the motif is fully deformable, the behaviour remains the same; this region affects only the sensitivity of the curve (Δε_max_/ΔT) and may slightly modify the global min/max values. (**E**) Generated function ${\rm{\varepsilon }}({{\rm{G}}}_{[\{{c}_{1},{c}_{2}\},3]}^{[1212]},{\rm T})$ for N = 4, N = 48 motifs as a function of temperature. (**F**) Emissivity as a function of the number of motifs for three different orientation permutations.

Figure 8a,b present group C[112], and three out of ten patterns with their limits. In Fig. 8c we present schematically the interaction of the motifs for three selected patterns with a {112} colour sequence and their influence on the overall thermal emission. If the motif is fully deformable, the behaviour remains the same; this region only affects the sensitivity of the curve (Δε_max_/ΔT), and may slightly modify the global min/max values, Fig. 8d. Figure 8d illustrates that the emissivity curve of more sensitive surfaces maintains the same shape. This indicates that the colour interactions overcome the interactions between the transformable areas of the motifs.

Finally, to exclude the energy, which is emitted from the edges of the pattern, we developed models with a different number of motifs, namely 4 < N < 65; then, the effective emissivity was calculated as a function of the temperature. Small deviations were presented between N = 4 and N = 64 motifs because of the minimization of edge effects (Fig. 8e,f).

### Material Structure and Fabrication

We can tile a surface using a combination of motifs, which form a pattern. The patterned surfaces have an overall area of (A = N × A_M_), where A_M_ is the region of the motif. The motif consists of a non-deformable region, the deformable region A_AM_ (depicted as yellow and green) and a region which may be either deformable or non-deformable. The higher the fraction *F*_*M*_ = *A*_*AM*_/*A*_*M*_ ≤ 1 and the ratio ε_1_/ε_2_ are—which correspond to the inner and outer surfaces, respectively—the higher the change of the emissivity (Δε_max_) (See Supplementary Information).

### Measurements and apparatuses

We used a comparative calorimetric method under vacuum to measure the effective emissivity (See Supplementary Information).

### Other measured and developed smart materials

We investigated a patterned surface which incorporated a ditranslational pattern with a rectangular lattice pattern (4 × 4 motifs) (See Supplementary Information).

### Data availability

The datasets generated during and/or analysed during the current study are available from the corresponding author on reasonable request.

## Electronic supplementary material

SUPPLEMENTARY INFORMATION

Supplementary Video S1

Supplementary Video S2.

Supplementary Video S3.

Supplementary Video S4.

Supplementary Video S5.

Supplementary Video S6.

Supplementary Video S7.
