# Supplementary material for: Smart patterned surfaces with programmable thermal emissivity and their design through combinatorial strategies
Source: Sci Rep. 2017 Oct 10;7:12908. doi: 10.1038/s41598-017-13132-6 (PMC5635011; doi:10.1038/s41598-017-13132-6)
Supplement: Supplementary file 1 — SUPPLEMENTARY INFORMATION [file 41598_2017_13132_MOESM1_ESM.pdf]

SUPPLEMENTARY INFORMATION – SI

Smart patterned surfaces with programmable thermal emissivity and their design through combinatorial strategies

N. Athanasopoulos[a,1], N. J. Siakavellas[a]
[a]Department of Mechanical Engineering & Aeronautics, University of Patras, 26500, Patras, Greece
[1]Corresponding author. Tel.:+306946630065; fax:+302610997241. E-mail address: nathan@mech.upatras.gr, nikos.athanasop@gmail.com (N. Athanasopoulos).

The following information is provided for the replication of numerical models and experimental results.

1. Numerical modelling details: COMSOL Multiphysics was used for the modelling of the coupled problem. All parametric models were executed on a powerful workstation, namely the Fujitsu CELSIUS R930 (two parallel processors, 24 cores) with two Intel Xeon E5-2697 v2 processors (2.70 GHz, 30 MB cache) Turbo Boost (256 GB RAM). Owing to the complexity of the problem, we developed patterned surfaces which can be modelled through 2D plain strain problems (Supplementary Videos S6 and S7). The 3D models may use more than 512 GB of RAM for a smart surface with 36 motifs because of the thermal radiation coupling. By solving the parametric models, we calculated the temperature field of the surface and the total hemispherical emissivity. All material properties and model dimensions are listed in Tables S1 and S2.

Table S1. Material Properties.

Emissivity, c_1	ε_1	0.95	-
Emissivity, c_2	ε_2	0.075	-
Thermal conductivity	k_1	155	W/mK
Thermal conductivity	k_2	0.4	W/mK
Heat capacity	cp_1	893	J/kg·K
Heat capacity	cp_2	2000	J/kg·K
Young Modulus	E_1	69	GPa
Young Modulus	E_2	0.5	GPa
Poisson	v_1	0.33	-
Poisson	v_2	0.4	-
Coefficient of thermal expansion	a_1	200	(10^{-6}) m/m°C
Coefficient of thermal expansion	a_2	23.2	(10^{-6}) m/m°C

To ensure that the models accurately predict the deformed geometry, several models were developed and compared with results from analytical solutions. The generalized solution for the analytical prediction of any multilayer material with rectangular geometry was expressed as a system of equations (47). The trimaterial which we used can be modelled using a simplified equation for the bilayer material owing to the very small thickness of the middle adhesive layer. The curvature of the solved models was compared with that of the analytical solutions for the bilayer material. The curvature of a bilayer material can be calculated as:

$$\kappa = \Delta T\left(a_1 - a_2\right)\frac{6E_1E_2h_1h_2\left(h_1+h_2\right)}{E_1^2h_1^4+4E_1E_2h_1^3h_2+6E_1E_2h_1^2h_2^2+4E_1E_2h_2^3h_1+E_2^2h_2^4}, \qquad (S1)$$

where κ is the curvature, (α_1, α_2) are the coefficients of thermal expansion, (E_1, E_2) are the moduli of elasticity, (h_1, h_2) are the layer thicknesses, and ΔT is the temperature difference. We compared the curvature of the geometrically non-linear models with that of the analytical solutions for different temperature levels. The results are listed in Table S3.

Table S2. Dimensions of the motifs and the patterns for the numerical modelling, for partially and fully deformable active regions.

		**Thickness = 125 µm Motifs, N = 4**		**Thickness = 25 µm Motifs, N = 4**	
		Case_1 (Partially deformable)	Case_2 (Fully deformable)	Case_1 (Partially deformable)	Case_2 (Fully deformable)
		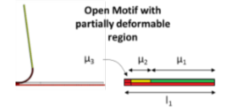	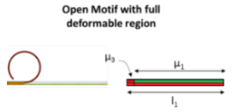	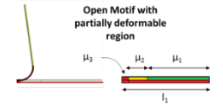	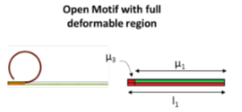
**l_1 (Length of the motif)**	mm	8.5	8.5	8.5	8.5
**μ_1 (Length of the non-deformable area)**	mm	6	0	6	0
**μ_2 (Length of the deformable area)**	mm	2	8	2	8
**μ_3 (Length of the non-deformable area)**	mm	0.5	0.5	0.5	0.5
**L (Total length of the patterned surface)**	mm	34	34	34	34
**A_A (Fraction of the active to the non-deformable area)**	-	0.25	1	0.25	1
**A_{AM} (Fraction of non-deformable area to the total area)**	-	0.94	0.94	0.94	0.94
**h_1 (Thickness of the 1st layer)**	µm	100	100	20	20
**h_2 (Thickness of the 2nd layer)**	µm	25	25	5	5

Table S3. Comparison of numerical and analytical results of the calculated curvature for the non-linear thermo-mechanical problem at different temperature levels.

	**Radius of curvature (m)**					
**Temperature change (°C)**	**Numerical Results**	**Analytical Solution**	**Relative Difference (%)**	**Numerical Results**	**Analytical Solution**	**Relative Difference (%)**
	**h_1=100 µm, h_2=25 µm**			**h_1 = 40 µm, h_2 = 10 µm**		
56.85	9.54	9.46	0.88	3.79	3.78	0.19
106.85	5.15	5.03	2.35	2.05	2.01	1.86
156.85	3.55	3.43	3.59	1.43	1.37	4.30

2. Material Structure and Fabrication: We can tile a surface using a combination of motifs, which form a pattern. The patterned surfaces have an overall area of ($A = N\times A_M$), where A_M is the region of the motif (depicted as red, green, and yellow). The motif consists of a non-deformable region (depicted as red), the deformable region A_{AM} (depicted as yellow and green) and a region which may be either deformable or non-deformable (depicted as green), (Fig. S1).

The higher the fraction $F_M = A_{AM}/A_M \leq 1$ and the ratio $\varepsilon_1/\varepsilon_2$ are—which correspond to the inner and outer surfaces, respectively—the higher the change of the emissivity ($\Delta\varepsilon_{max}$).

The deformable regions are very responsive to temperature, presenting extremely large deformations (Fig. S1f; Video S4). These materials are similar to the "4D-biomimetic materials", which become activated in proportion to the stimulus (humidity[37,39-40] or temperature) and can be manufactured via low-cost techniques. The mismatch of the coefficient of thermal expansion (CTE) between the

anisotropic layers creates materials that are very sensitive in temperature and alter their shape drastically owing to the developed internal stresses and their anisotropic nature. The sequence of the layers and the materials of the non-deformable and deformable regions that were used for the development of various patterned surfaces are presented in Fig. S2 & Table S4.

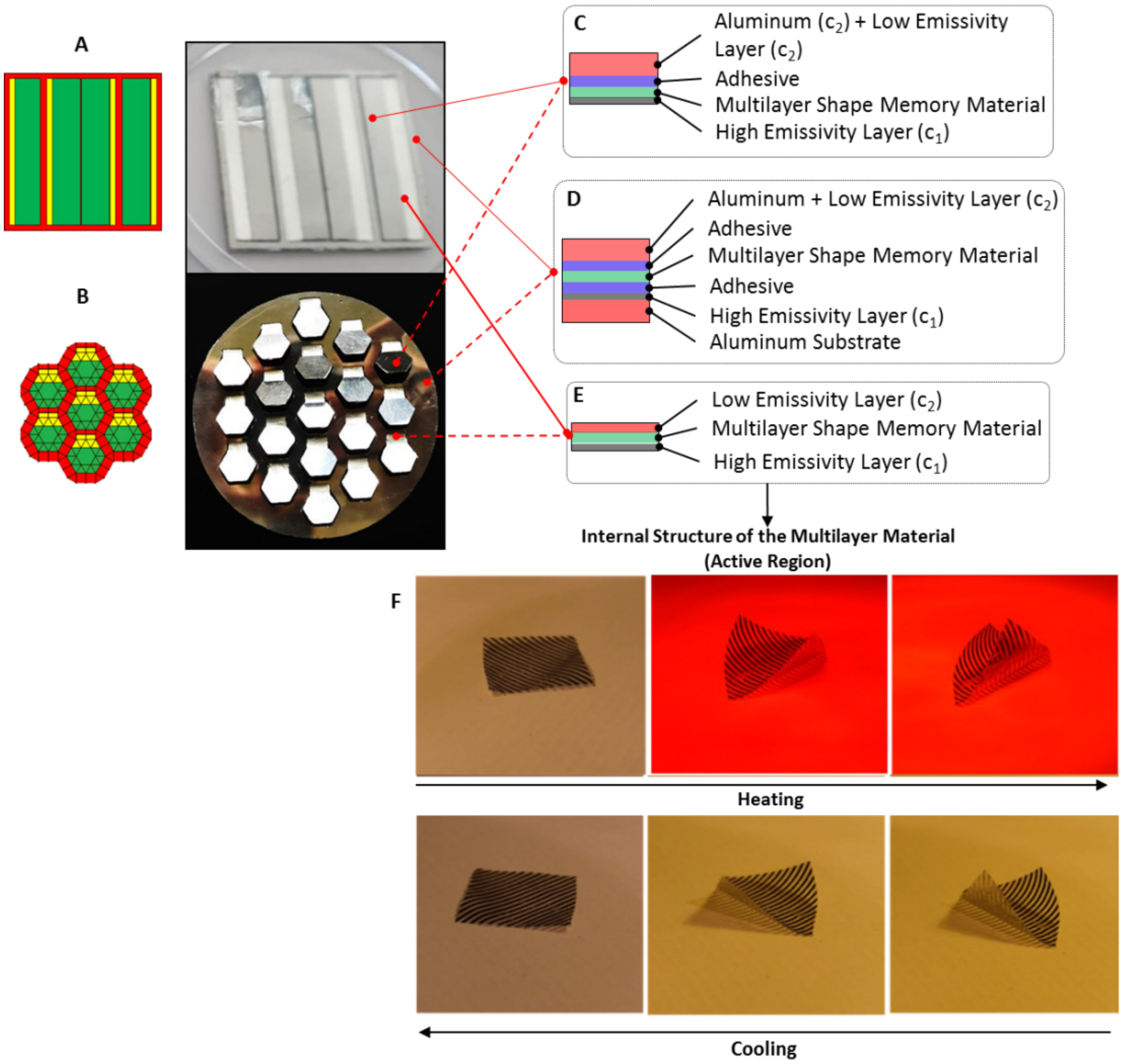

Fig. S1. Structure of the smart patterned surfaces. (A,B) Smart patterned surfaces with variable emissivity. (C-E) Deformable and non-deformable multilayer structure. (F) Multilayer shape memory material (Movie S4).

A two-component adhesive for low-energy plastics (Methacrylat- and Amine-based resins) was mixed with 1% MWCNTs (high emissivity > 0.9), and was applied on the oriented PE. An aluminium foil was applied on the adhesive, and was pressed under vacuum. The aluminium strips (20 μm thick) were formed using a chemical etching technique through ferric chloride solution at 40 ºC for 30–40 min. A thinner-component adhesive and an aluminium film of 4-5 μm (emissivity ≈ 0.08) were applied and pressed under vacuum. One more adhesive film was applied over the aluminium film, together with a thicker polished aluminium sheet (125 μm), and was pressed. The motifs were formed through the etching technique using a ferric chloride solution at 40 ºC for 150–160 min. The overall surface were immersed into an acetone bath for 2 h in order to remove the remaining adhesive, and a CNC laser was used to finalize the formation of the motifs. Finally, the patterned surface was placed over an aluminium substrate with a high-emissivity coating (graphite-based).

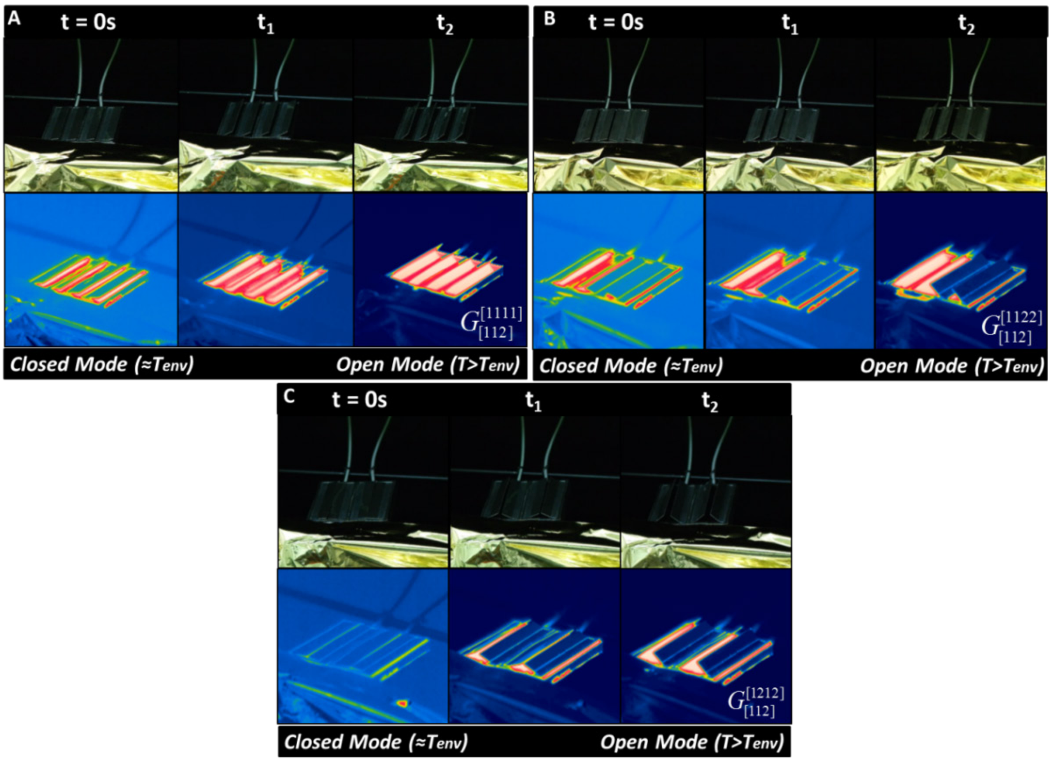

Fig. S2. The developed studied surfaces, incorporating rectangular motifs on a strip with dimensions of (54 × 54 mm), and thermographic images. (A) Pattern $G_{[112]}^{[1111]}$ (p111). (B) Pattern $G_{[112]}^{[1122]}$ (pm11). (C) Pattern $G_{[112]}^{[1212]}$ (pm11) (Movie S5).

Table S4. Internal structure of the multilayer patterned surfaces at different regions.

	**Deformable Region (Yellow)**	**Non-deformable Region (Red)**	**Deformable or Non-deformable Region (Green)**
1.	Oriented PE	Aluminium substrate	Oriented PE
2.	Adhesive	Graphite coating	Adhesive
3.	Aluminium strips	Oriented PE	Aluminium strips
4.	Aluminium film (5 µm)	Adhesive	Aluminium film
5.		Aluminium strips (%)	Adhesive
6.		Aluminium film	Polished Aluminium
7.		Adhesive	
8.		Polished Aluminium	

3. Detailed description of the total hemispherical emissivity measurements and apparatus: We employed a comparative calorimetric method under vacuum to measure the effective emissivity of the smart surfaces higher than the ambient temperature. We placed a black matt surface (ε_{black} = 0.95) on the aluminium thermal pad, which was then placed into the vacuum chamber; we measured the steady state temperature (T_{black} = T_i) as a function of the applied power ($\left. Q_{black}^{electrical} \right|_T$) at a certain medium vacuum level (12 ± 0.2 Pa) and ambient temperature ($T_\infty \approx$ 19.8 °C). The vacuum and the outer temperature remained constant for 200 min prior to starting each experiment. Each temperature level (T_i = 30 °C, 40 °C, 50 °C, 60 °C, 70 °C, 80 °C, 90 °C, and 100 °C) was reached; the steady-state was maintained for at least 30 min to ensure that there would be no temperature change versus time (T = $T_{Steady\text{-}state}$ ± 0.01 °C). While maintaining all aforementioned parameters constant, we placed the smart patterned surface on the aluminium thermal pad inside the vacuum chamber; we then identified the required applied power ($\left. Q_{smart}^{electrical} \right|_T$) for each aforementioned temperature level (T_{smart} = T_i). The effective emissivity was determined as a function of temperature through the following equation.

$$\left.\begin{array}{l} Q_{black}^{electrical}\Big|_T = Q_{black}^{rad} + \left(Q_{silver}^{rad} + Q_{black}^{losses}\right) \\ Q_{smart}^{electrical}\Big|_T = Q_{rad}^{smart} + \left(Q_{silver}^{rad} + Q_{black}^{losses}\right) \end{array}\right\} \overset{T_{smart}=T_{black}=T}{\Rightarrow} Q_{black}^{electrical}\Big|_T - Q_{black}^{rad} = Q_{smart}^{electrical}\Big|_T - Q_{smart}^{rad} \Rightarrow$$

$$\Rightarrow Q_{smart}^{rad} = Q_{smart}^{electrical}\Big|_T - Q_{black}^{electrical}\Big|_T + Q_{black}^{rad} \Rightarrow \varepsilon_{eff}^{smart} = \frac{VI^{smart} - VI^{black} + \varepsilon_{black}\sigma A\left(T^4 - T_\infty^4\right)_{black}}{\sigma A\left(T^4 - T_\infty^4\right)_{smart}} \qquad (S2)$$

We recorded and compared the measurements of (T, T_∞, V, I) for each effective emissivity value with those of the black coating. It is highly important that $\left(T^4 - T_\infty^4\right)_{black} \approx \left(T^4 - T_\infty^4\right)_{smart}$ between the two comparative measurements. Additionally, we recorded and compared the resistance of the overall circuit with the previous measurements to avoid uncertainties and potential internal faults. This method is very sensitive in temperature levels near the ambient temperature owing to the 4th power of the temperature. Small deviations may result to large discrepancies.

Thermal pad and measurement acquisition: We used a flexible silicon heater pad (3 Watts) to control the temperature of the smart surfaces (Fig. S3b). We connected the silicon heater pad using two highly conductive copper wires (6 mm^2) with a direct current (DC) voltage power supply. Voltage was applied on the thermal pad owing to the Joule effect, resulting in a uniform temperature increase on the surface. We attached the upper side of the heater pad to an aluminium flat plate of a 1.5 mm thickness. The aluminium sheet was covered with four layers of black matt paint, with an emissivity of ≈ 0.95. The opposite side of the pad was covered with a grooved aluminium plate. We attached a pure highly polished silver sheet with a thickness of 0.3 mm at the lower and external surface of the aluminium thermal pad to prevent heat transfer through radiation as much as possible. Two thin K-thermocouples were placed inside the 1st aluminium sheet. The supplied electrical power must be accurately controlled as a function of the steady-state temperature of each sample. For this reason, we used a stabilised DC power supply (TTi QPX1200SP Bench Power Supply) to accurately regulate the voltage (accuracy Voltage–resolution Voltage - 1 mV). We measured the applied voltage with a multimeter (GW Instec, GDM-8251A), with an accuracy of ±(0.012% rdg + 5 digits). The current was measured with accuracy of ±100 μA.

Temperature acquisition and thermocouples: We placed two K-thermocouples, specially designed for vacuum conditions, inside the thermal pad. We placed the tip of the thermocouple 0.1 mm below the middle of the outer surface of the material to measure the temperature near the radiative surface, and to minimise measurement uncertainties caused by the temperature gradient through the thickness. The junctions of the thermocouples were covered with an ultrathin electrical epoxy-based insulating film. The vacuum chamber operated near room temperature; we continuously recorded the temperature of the inner surface (T_∞) using an array of six thermocouples. The thermocouple arrays measured the temperature of the internal wall of the vacuum chamber (0.1 mm above the internal black surface). The measurements were continuously recorded with a 500 ms sampling rate, and stored in a PC via a calibrated USB data acquisition hardware (Picolog TC-08). All measurements were obtained once steady-state conditions were reached.

Vacuum Chamber: To avoid measurement discrepancies, we used a steel vacuum chamber; we coated all inner wall surfaces with three layers of black matt paint, with an emissivity of approximately ε ≈ 0.95, to create a large black-body cavity, and to ensure that the reflections would be negligible (Fig. S3a). We realised this black-body effect by employing a highly absorbing surface, by making the surface area considerably larger than that of the specimen, and by avoiding any external energy radiation sources. The diameter of the chamber was (D = 0.36 m) and the weight was 40 kg. The thermal pad and the investigated materials were located near the centre of the vacuum chamber. The

relationship between the vacuum chamber size and its required surface emittance was estimated from the following equation for the shape factor of a grey body, for a surface completely enclosed by another surface. To ensure that the external environment acts as a black body, the following condition must apply (48).

$$\frac{1}{\varepsilon_{material}} >> \frac{A_1}{A_2}\left(\frac{1}{\varepsilon_{chamber}} - 1\right) \qquad (S3)$$

This condition can be satisfied for all possible values of specimen emittance by using an apparatus design in which A_1/A_2 = (area of pad)/(internal area of vacuum chamber). The patterned surface may change its emissivity from very low to very high emissivity. The lower surface of the thermal pad was covered with a pure and highly polished silver foil (thickness of 0.3 mm). Assuming that the upper surface of the patterned surface has a maximum total hemispherical emissivity of 0.9, and that the lower surface of the pad has an emissivity value of less than 0.05, we may expect that the vacuum chamber approximately behaves as a large black body, Eq. 16. For the open and closed mode of the patterned surface, the calculated ratios are the following.

$$\frac{1/0.9}{\frac{A_{pad}}{A_{chamber}}\left(\frac{1}{0.95} - 1\right)} = 2.5\times10^3 \quad and \quad \frac{1/0.075}{\frac{A_{pad}}{A_{chamber}}\left(\frac{1}{0.95} - 1\right)} = 30.4\times10^3$$

The lower surface of the thermal pad (silver side of the thermal pad) has a ratio equal to the following:

$$\frac{1/0.035}{\frac{A_{pad}}{A_{chamber}}\left(\frac{1}{0.95} - 1\right)} = 65.2\times10^3$$

For all cases, the calculated ratios are very high, thus ensuring that the chamber behaves as a black body. Vacuum was achieved by using two oil vacuum pumps (2-stage, 1.99×10^{-2} mbar) with an overall flow rate equal to 284 L/min; the vacuum level was measured with a Supco VG64 digital vacuum gauge.

Temperature Uniformity: The temperature uniformity over the heater pad (Fig. S3b) ensures that the entire surface radiates energy uniformly. In this case, we assumed that the temperature was the same for the entire surface, and could be expressed as a simple parameter, namely T = T(x,y) for every (x, y). Two different comparisons were carried out: **i.** comparison between two thermocouples, and **ii.** temperature uniformity analysis using thermography. The temperature deviation between the two thermocouples during the heating at the lower and higher temperature level is presented in Fig. S3c. The relative difference between the two temperature measurements during the heating stage or at the steady state was less than 0.43% (Fig. S3c). In both setups, an infrared (IR) camera was located above the samples, and recorded the emitted radiation during heating. The IR camera was FLIR SC660, which has a high resolution pixel detector of 640 × 480 pixels, and has a thermal sensitivity of ≤ 45 mK. The thermal camera images prove that the thermal pad was uniformly heated (Fig. S3d). After analysing the area, we calculated that the average temperature was 92.5 °C with a standard deviation of 0.5, whereas the thermocouple indicated a temperature of 92.7 °C. For the second temperature level, the average temperature was 30.4 °C with a standard deviation of 0.1, whereas the thermocouple indicated a temperature of 30.4 °C. These very small discrepancies did not affect the obtained experimental measurements.

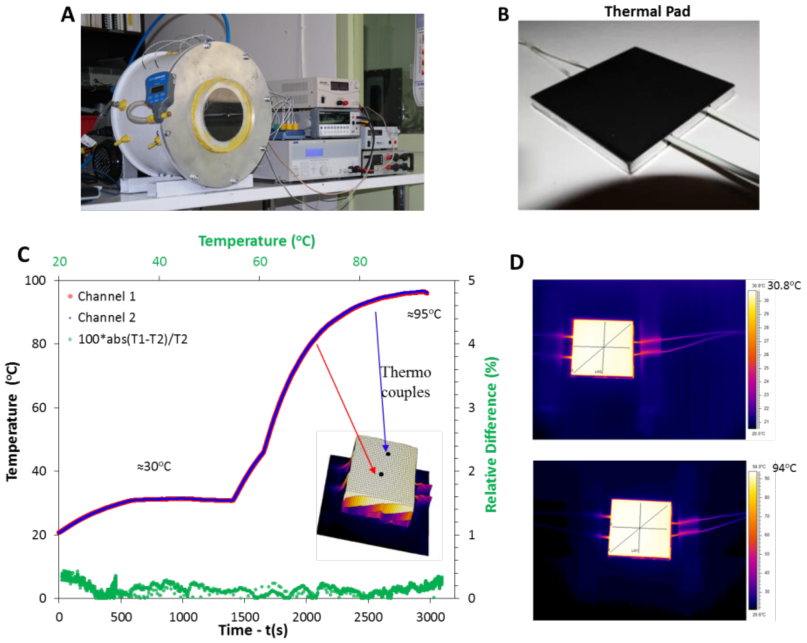

Fig. S3. Experimental methodology and apparatus. (A) Vacuum chamber apparatus. (B) Thermal pad device for the activation of the patterned surfaces. (C) Temperature response of the measurement points under random power supply level and relative difference. (D) Temperature field obtained from thermography near the lowest and the highest temperature level.

4. Other measured and developed smart materials. We investigated a patterned surface which incorporated a ditranslational pattern with a rectangular lattice pattern (4 × 4 motifs). The emissivity increased within the temperature range of 20 °C to 57 °C. For higher temperatures, the emissivity remained almost constant. The developed surfaces could changed their emissivity $\Delta\varepsilon = 0.38$ within a very small temperature deviation ($\Delta T = 37$ °C), (Fig. S4).

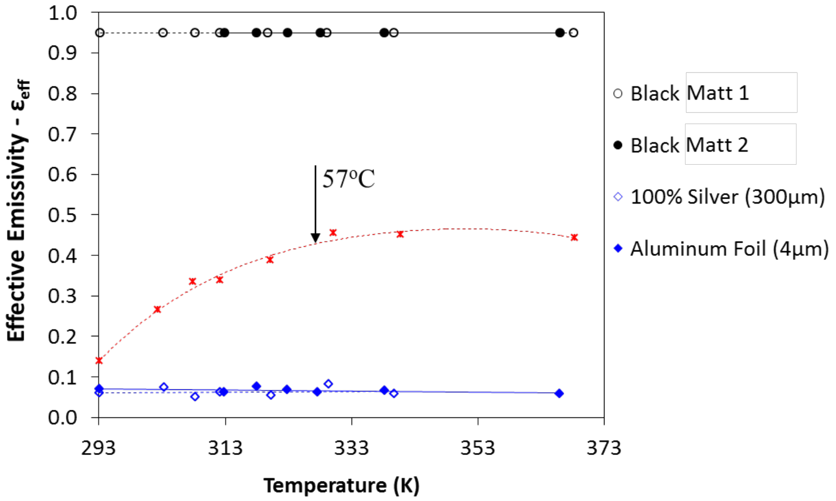

Fig. S4. Developed patterned surface (p1(r1)) with C[{1,2},3], N = 4 × 4 motifs. Total hemispherical emissivity as function of temperature.

5. Supplementary Videos

Supplementary Video S1. Developed pattern surface with low to high emissivity and thermographic images during heating (the surface emit more in higher temperature).

Supplementary Video S2. Developed pattern surface with high to low emissivity and thermographic images during heating (the surface emit less in higher temperature). Despite the fact that all surfaces are

at a similar temperature, the thermal camera captures a temperature field that it is not ‘correct’ owing to the very low emissivity of the outer material of the surface (thermal camera set to $\varepsilon \approx 0.95$).

Supplementary Video S3. Developed pattern surface on a hexagonal lattice, C[112] from high to low emissivity (open to closed state), and thermal cycles under infrared light.

Supplementary Video S4. Shape transformation of the multilayer material during heating and cooling. The oriented polyethylene and the aluminium strips present anisotropic thermo-mechanical properties.

Supplementary Video S5. Shape transformation of the developed surfaces, incorporating rectangular motifs on a strip with dimensions of (54 × 54 mm), and thermographic images. (A) Pattern $G_{[112]}^{[1111]}$ (p111). (B) Pattern $G_{[112]}^{[1122]}$ (pm11). (C) Pattern $G_{[112]}^{[1212]}$ (pm11).

Supplementary Video S6. Transient thermo-mechanical models for pattern $G_{[112]}^{[1111]}$ with fully deformable motifs (8 mm), and calculation of the ambient view factor during the heating stage. Blue line depicts the external surface (position: p3), red line depicts the internal lower surface (position: p1), green line depicts the internal middle surface (position: p2).

Supplementary Video S7. Transient thermo-mechanical models for pattern $G_{[112]}^{[1111]}$ with partially deformable motifs (2 mm), and calculation of the ambient view factor during the heating stage.
Blue line depicts the external surface (position: p3), red line depicts the internal lower surface (position: p1), green line depicts the internal middle surface (position: p2).

Supplementary References

47. Vasudevan M, Johnson W. On multi-metal thermostats. *Appl Sci Res Sect B* 9(6):420–430 (1963).

48. ASTM C 835 – 95. Standard Test Method for Total Hemispherical Emittance of Surfaces From 20 to 1400 °C (1999).
